# The Evolution of Artificial Intelligence in Ocular Toxoplasmosis Detection: A Scoping Review on Diagnostic Models, Data Challenges, and Future Directions

**DOI:** 10.3390/idr17060148

**Published:** 2025-12-08

**Authors:** Dodit Suprianto, Loeki Enggar Fitri, Ovi Sofia, Akhmad Sabarudin, Wayan Firdaus Mahmudy, Muhammad Hatta Prabowo, Werasak Surareungchai

**Affiliations:** 1 Doctoral Program in Medical Science, Faculty of Medicine, Universitas Brawijaya, Malang 65145, Indonesia; dodit.suprianto@polinema.ac.id; 2 Department of Electrical Engineering, Politeknik Negeri Malang, Malang 65141, Indonesia; 3 Department of Clinical Parasitology, Faculty of Medicine, Universitas Brawijaya, Malang 65145, Indonesia; 4 AIDS, Toxoplasma, Opportunistic Disease and Malaria (ATOM) Research Group, Faculty of Medicine, Universitas Brawijaya, Malang 65145, Indonesia; 5 Department of Ophthalmology, Faculty of Medicine, Universitas Brawijaya, Dr. Saiful Anwar General Hospital, Malang 65111, Indonesia; dr.ovisofia@ub.ac.id; 6 Department of Chemistry, Faculty of Science, Universitas Brawijaya, Malang 65145, Indonesia; sabarjpn@ub.ac.id; 7 Department of Informatics Engineering, Faculty of Computer Science, Universitas Brawijaya, Malang 65145, Indonesia; wayanfm@ub.ac.id; 8 Department of Pharmacy, Faculty of Mathematics and Natural Sciences, Universitas Islam Indonesia, Yogyakarta 55584, Indonesia; hatta.prabowo@uii.ac.id; 9 School of Bioresources and Technology, King Mongkut’s University of Technology Thonburi, Bangkok 10140, Thailand; werasak.sur@kmutt.ac.th

**Keywords:** Ocular Toxoplasmosis, deep learning, diagnosis, fundus photography, clinical translation

## Abstract

Ocular Toxoplasmosis (OT), a leading cause of infectious posterior uveitis, presents significant diagnostic challenges in atypical cases due to phenotypic overlap with other retinochoroiditides and a reliance on expert interpretation of multimodal imaging. This scoping review systematically maps the burgeoning application of artificial intelligence (AI), particularly deep learning, in automating OT diagnosis. We synthesized 22 studies to characterize the current evidence, data landscape, and clinical translation readiness. Findings reveal a field in its nascent yet rapidly accelerating phase, dominated by convolutional neural networks (CNNs) applied to fundus photography for binary classification tasks, often reporting high accuracy (87–99.2%). However, development is critically constrained by small, imbalanced, single-center datasets, a near-universal lack of external validation, and insufficient explainable AI (XAI), creating a significant gap between technical promise and clinical utility. While AI demonstrates strong potential to standardize diagnosis and reduce subjectivity, its path to integration is hampered by over-reliance on internal validation, the “black box” nature of models, and an absence of implementation strategies. Future progress hinges on collaborative multi-center data curation, mandatory external and prospective validation, the integration of XAI for transparency, and a focused shift towards developing AI tools that assist in the complex differential diagnosis of posterior uveitis, ultimately bridging the translational chasm to clinical practice.

## 1. Introduction

Ocular Toxoplasmosis (OT) represents the leading infectious cause of posterior uveitis worldwide, affecting an estimated one-third of the global population chronically infected with *Toxoplasma gondii*. The disease demonstrates significant geographic variation in severity, with particularly aggressive manifestations in South America due to highly virulent parasite genotypes [1]. Its recurrent nature leads to sight-threatening complications—including retinal detachment, choroidal neovascularization, and irreversible chorioretinal scarring—that collectively contribute to permanent visual impairment in a significant proportion of patients [2,3]. While advanced imaging modalities like optical coherence tomography angiography (OCTA) have revolutionized our understanding of OT by revealing profound retinal and choroidal involvement, these very advances have also underscored the extreme complexity of its pathological changes, which demand expert interpretation [4]. This substantial global health burden, characterized by significant visual morbidity and intricate diagnostic challenges in atypical cases, underscores the urgent need for innovative approaches to enhance diagnostic precision and improve patient outcomes.

The diagnostic pathway for OT remains challenging due to several interconnected factors. First, while the diagnosis of OT integrates objective serological tests with clinical assessment, these tests do not always provide conclusive results [5]. Serological tests can indicate a latent infection rather than an active one, results may be false-negative in immunocompromised individuals, and molecular techniques like PCR can exhibit low sensitivity. Consequently, in atypical cases, diagnosis often relies heavily on the subjective interpretation of clinical manifestations and multimodal imaging by specialist clinicians, creating an inherent variability in diagnostic accuracy [6]. Second, atypical OT demonstrates phenotypic overlap with other necrotizing retinochoroiditides, including acute retinal necrosis, syphilitic uveitis, and tuberculous retinitis, particularly complicating differential diagnosis in resource-limited settings where access to advanced testing is constrained [7,8]. While OCTA provides unprecedented detail of retinal and choroidal changes, including microvascular alterations and ischemia patterns, its interpretation requires specialized expertise that is frequently unavailable in high-burden regions [4]. Furthermore, atypical presentations such as papillitis without vitritis or unilateral neuroretinitis further complicate the diagnostic process, potentially leading to treatment delays and irreversible visual damage [9]. These challenges collectively create a critical diagnostic bottleneck that necessitates innovative solutions to standardize interpretation and improve accessibility.

Artificial intelligence (AI), particularly deep learning (DL), has emerged as a transformative force in medical imaging, demonstrating remarkable success in ophthalmology by diagnosing sight-threatening diseases such as diabetic retinopathy, glaucoma, and age-related macular degeneration with accuracy comparable to experts [10,11,12]. This proven capability to analyze complex ophthalmic data, from fundus photographs to OCT scans, offers considerable promise for addressing the longstanding diagnostic challenges in Ocular Toxoplasmosis (OT). The application of AI to automate image analysis, quantify pathological features, and provide objective decision support could potentially mitigate the current reliance on expert clinicians, reduce subjectivity in interpretation, and improve the differentiation of OT from other necrotizing retinochoroiditides. Initial proof-of-concept studies have indeed demonstrated the potential of convolutional neural networks (CNNs) to achieve high diagnostic accuracy in identifying OT lesions.

However, the current body of literature reflects a nascent and highly heterogeneous field. Research efforts are characterized by divergent approaches across key dimensions: utilizing varied imaging modalities (from fundus photography to advanced OCTA), pursuing different algorithmic tasks (binary classification, differential diagnosis, lesion segmentation), and employing diverse model architectures with substantially different training methodologies. This methodological heterogeneity, combined with the field’s emergent nature, has created a fragmented evidence base that cannot be adequately synthesized through a traditional systematic review focused solely on efficacy metrics.

Therefore, a scoping review methodology is uniquely appropriate for this domain. This approach allows for the systematic mapping of the current technological landscape without imposing artificial homogeneity on inherently diverse studies. It enables the inclusion of the full spectrum of research activity, from technical validations to early clinical applications. The primary objectives of this review are consequently conceptual and exploratory: to identify key methodologies and applications; to map the evidence according to central themes such as model types, data sources, and validation strategies; to critically examine persistent gaps concerning data scarcity, external validation, and clinical integration; and ultimately, to establish a foundational framework that directs future research priorities. By doing so, this review aims to provide an essential roadmap for guiding the development of AI in OT from exploratory innovation toward robust, clinically translatable solutions.

Guided by the need to map this heterogeneous and emerging field systematically, the primary objective of this scoping review is to comprehensively synthesize and critically examine the current body of literature on the application of artificial intelligence for the diagnosis of Ocular Toxoplasmosis. This review seeks to move beyond a mere cataloguing of studies to construct an integrated landscape that elucidates the interplay between technological innovation, data infrastructure, and clinical translation. Our synthesis will specifically focus on characterizing the developmental trajectory of diagnostic models, auditing the data paradigms upon which they are built, and evaluating their readiness for integration into clinical pathways.

To achieve this objective and ensure a structured and impactful inquiry, this review is guided by the following specific research questions:

- State of the Evidence. What is the scope and nature of the existing evidence regarding the development and validation of AI models for the diagnosis of Ocular Toxoplasmosis?
- The Data Landscape and Its Challenges. What are the characteristics—including size, source, modality (particularly the incorporation of OCTA), and availability—of the datasets used to train and validate these AI models?
- Pathways to Clinical Translation. What is the state of progress regarding the clinical validation and implementation readiness of these AI tools?

## 2. Materials and Methods

### 2.1. Study Design

This study utilized a scoping review framework [13,14]. Two researchers independently conducted the database search and screened articles for relevance and inclusion. Any disagreements were resolved through consensus. This scoping review was conducted in accordance with the Preferred Reporting Items for Systematic Reviews and Meta-Analyses (PRISMA) 2020 statement [15].

### 2.2. Identification of Relevant Studies

A systematic literature search was conducted across PubMed, Scopus, and IEEE Xplore databases for studies published between January 2010 and August 2025. The search employed tailored search strings related to Ocular Toxoplasmosis, artificial intelligence, and diagnostic applications. The core query incorporated key terms such as (“*Ocular Toxoplasmosis*” *OR* “*Toxoplasmic retinochoroiditis*”) *AND* (“*artificial intelligence*” *OR* “*deep learning*” *OR* “*machine learning*”) *AND* (*diagnos OR detect*) to ensure a focused retrieval of relevant studies.

The initial search yielded 139 records (PubMed = 50, Scopus = 79, IEEE = 10). These records were processed through Rayyan.ai software (Cloud Version, Rayyan Systems, Inc., Cambridge, MA, USA), where 34 duplicates were removed and 5 records were marked as inaccessible from the database. The remaining 102 records underwent a title and abstract screening on Rayyan.ai’s collaborative platform, resulting in the exclusion of 70 irrelevant studies. Following a full-text assessment of the remaining 32 articles, ten were excluded due to non-diagnostic focus (*n* = 5), unclear methodology (*n* = 2), or poorly defined objectives (*n* = 1). Ultimately, 22 studies were included in the systematic review.

### 2.3. Selection of Articles

#### 2.3.1. Data Screening

**Figure 1:**
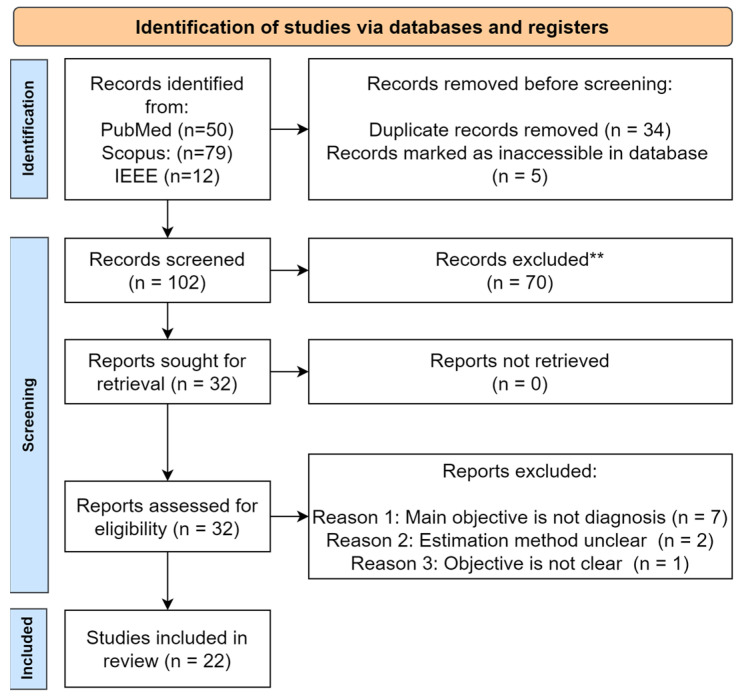
PRISMA flow diagram for selection of articles for the proposed scoping review.

Following manual scrutiny against the inclusion criteria, 22 publications were selected, and the article selection process is illustrated in Figure 1.

#### 2.3.2. Inclusion and Exclusion Criteria

Studies were included if they: (1) developed/validated AI/ML/DL models for OT diagnosis, segmentation, or classification; (2) involved human subjects with suspected/confirmed OT; (3) applied AI to retinal imaging (fundus, OCT, or OCTA); and (4) reported quantitative performance metrics (accuracy, sensitivity, specificity, AUC, Dice, or IoU).

Studies were excluded if they: (1) were reviews, editorials, conference abstracts, small case reports (<5 cases), protocols, or non-peer-reviewed publications; (2) focused on non-OT conditions, animal models, or simulations without clinical imaging; (3) used AI only for preprocessing or non-diagnostic tasks; (4) lacked quantitative performance metrics; or (5) were unavailable in English despite access efforts.

### 2.4. Data Charting

Each study was systematically summarized to extract key information on the techniques employed, datasets utilized, evaluation methodologies, and primary outcomes. Subsequently, the publications were classified and analytically compared based on their detection modality or technical approach using Microsoft Excel and Mendeley Cite (Version 1.19.8, Mendeley Ltd., London, UK).

### 2.5. Summarizing and Reporting the Results

The extracted data were synthesized and summarized to provide a comprehensive mapping of the evidence, explicitly designed to answer the three research questions guiding this scoping review.

To address research question 1 (State of the Evidence), the results were organized to delineate the scope and nature of the included studies. This involved charting the chronological and geographical distribution of research, cataloging the various AI model architectures (e.g., CNNs, SVMs, Transformers, hybrid models), and classifying the primary diagnostic tasks undertaken (e.g., binary classification, multiclass differentiation of disease activity, lesion segmentation).

In response to research question 2 (The Data Landscape and its Challenges), the synthesis focused on quantitatively and qualitatively summarizing the characteristics of the datasets employed across all studies. This included documenting dataset sizes, origins (single-center vs. multi-center), imaging modalities (e.g., fundus photography, OCT, OCTA), and availability (public vs. private). The results were then analyzed to identify and report the most frequently cited limitations and challenges in data curation, such as small sample sizes, significant class imbalance, and the lack of public datasets.

To answer the research question (Pathways to Clinical Translation), the findings were summarized to evaluate the clinical readiness of the proposed AI tools. This encompassed an analysis of the validation rigor employed (e.g., internal cross-validation vs. external validation on independent datasets), the exploration and integration of explainable AI (XAI) methods, and the extent to which studies discussed practical implementation into clinical diagnostic pathways.

The synthesized results are reported narratively in the subsequent sections and are further organized into structured tables and Figures. These summaries provide a clear foundation for interpreting the current state of the field, its predominant challenges, and its future trajectory, facilitating the identification of key gaps and informing recommendations for subsequent research and development.

### 2.6. Protocol and Registration

This scoping review was conducted based on an a priori protocol that defined its objectives and methodology. This scoping review was not registered. The methodology is reported in full in this manuscript to ensure transparency and reproducibility (Supporting information Table S1).

## 3. Results

This scoping review synthesized findings from 22 studies dedicated to the development and validation of artificial intelligence (AI) models for diagnosing Ocular Toxoplasmosis (OT) (see Table 1 for a summary).

### 3.1. Subsection

**Figure 2:**
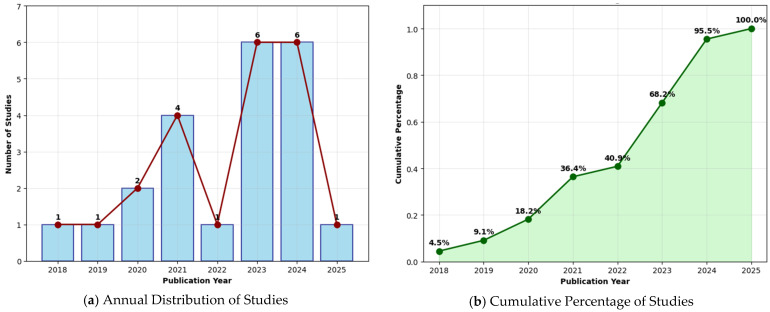
Chronological Distribution of AI Studies in Ophthalmology (2018–2025). (**a**) Annual Distribution of Studies, (**b**) Cumulative Percentage of Studies.

Analysis of the publication timeline reveals a field that was nascent before 2020 but has since entered a phase of rapid acceleration. As detailed in Table 2 and Figure 2, only two studies were published in 2018 and 2019 combined. A significant surge began in 2021 (*n* = 4), rising sharply to a peak of six studies in 2023. This high level of activity has been sustained, with another six studies published in 2024 and three studies already in 2025. In total, 20 of the 22 included studies (90.9%) were published from 2020 onwards, underscoring the rapidly growing academic and clinical attention toward AI-assisted OT diagnosis in recent years [16,22,23,24,27,28,29].

**Figure 3:**
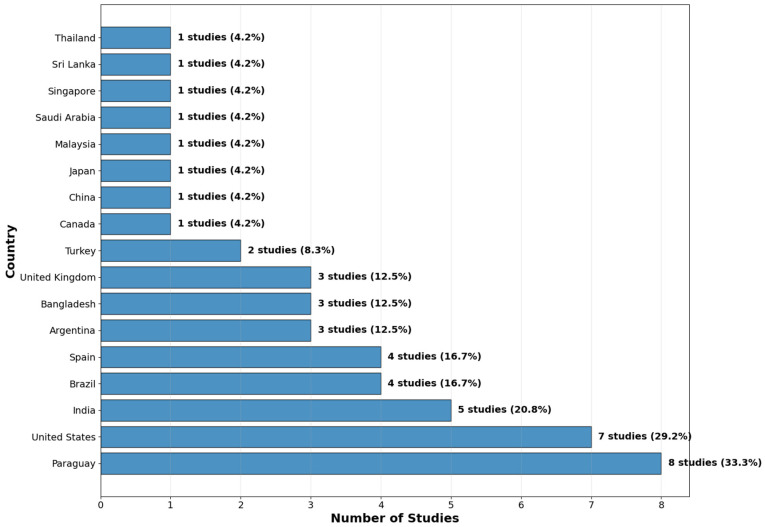
Country Distribution of AI Studies in Ophthalmology (2018–2025).

Geographical analysis of author affiliations revealed contributions from researchers in 17 distinct countries, indicating a strong and truly global interest. As detailed in Table 3, Paraguay was associated with the highest number of studies (*n* = 6), followed by the United States (*n* = 5) and Spain (*n* = 4). Brazil and India were also highly represented (*n* = 3 each). This distribution highlights a significant and impactful contribution from research groups in regions with high clinical relevance and OT prevalence [33,34,36], as well as from major technological hubs. It is critical to note that these Figures account for all authors’ nationalities per study; the total number of country affiliations (*n* = 34) exceeds the number of included studies (*n* = 22) due to frequent and robust international collaborations, particularly between South American and European institutions (see Figure 3).

Methodologically, the landscape is dominated by experimental AI model development studies (*n* = 19, 86%), most of which applied retrospective designs and utilized existing datasets of fundus images. Common validation strategies included k-fold cross-validation and train-test splits, reflecting an early proof-of-concept phase in model deployment. Only a small number of studies incorporated clinical perspectives or expert review, and even fewer applied external validation, illustrating a gap in translational readiness [16,17,21].

**Figure 4:**
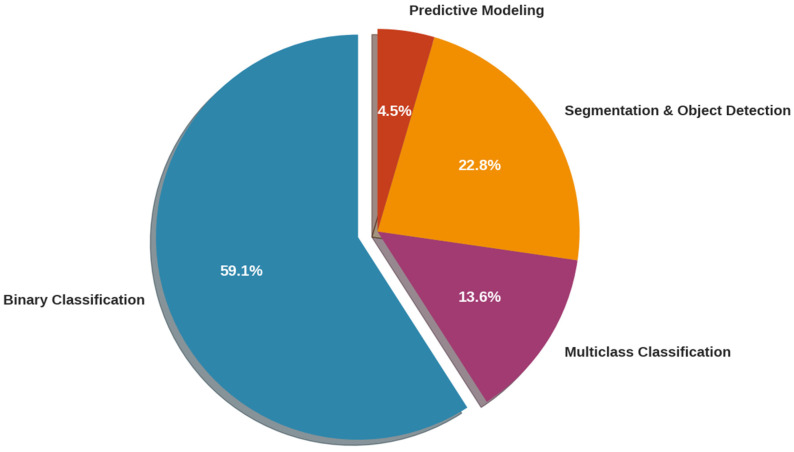
Primary Diagnostic Tasks.

The primary diagnostic task addressed by AI models was binary classification (e.g., distinguishing healthy and infected eyes), which appeared in 13 studies (59.1%). More complex multiclass classification, such as differentiation between active, inactive, and healthy conditions, was implemented in a smaller subset (3 studies, 13.6%). Segmentation and object detection tasks—focusing on lesion localization—were similarly limited but demonstrate important directionality toward spatial reasoning. A rare but notable case applied AI to predictive modeling of disease recurrence, using clinical data rather than imaging [18,22,26] (see Figure 4).

**Figure 5:**
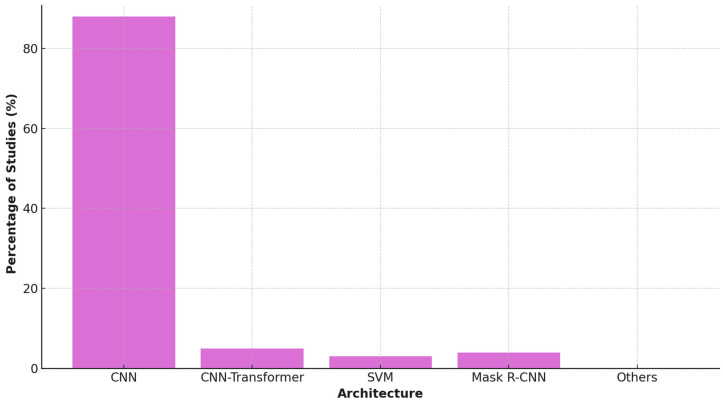
AI Architecture Usage in Studies.

In terms of technology, Convolutional Neural Networks (CNNs) were the predominant architecture (used in 19 studies, 86%), often leveraging transfer learning from pretrained backbones like VGG16, ResNet, and InceptionV3 to address data scarcity. More recent studies began to explore hybrid architectures, including CNN-Transformer models (e.g., RetinaCoAt), AutoML platforms (e.g., Google Cloud Vertex AI), and support vector machines (SVMs) using deep feature embeddings. Instance segmentation models such as Mask R-CNN were also introduced to improve lesion delineation accuracy in fundus images [24,25,28] (see Figure 5).

The predominance of specific architectures reflects pragmatic adaptations to the challenges of OT data. While standard CNNs like VGG16 provide a strong baseline, their performance can be limited on small datasets. The use of ResNet models [33,36] offered a key advantage through residual connections, which mitigate the vanishing gradient problem and enable more effective training of deeper networks—a crucial benefit given the limited data available for OT. In contrast, the emergence of more complex architectures like Transformer-based models [24] is promising but faces the inherent constraint of requiring vast amounts of data, which are not yet available for OT. This suggests that currently, architectures with built-in efficiency and robustness to overfitting, such as ResNet, are better suited for the OT domain than data-hungry state-of-the-art models. A summary of this comparative analysis, detailing the advantages, limitations, and clinical implications of the predominant architectures, is provided in Table 4.

### 3.2. Methodological Evolution and Study Characteristics

**Figure 6:**
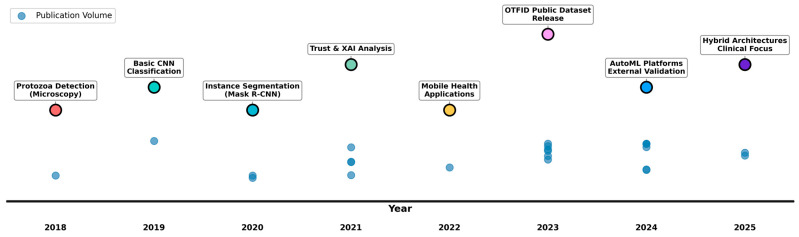
Conceptual Evolution of AI Research in Ocular Toxoplasmosis Diagnosis. This integrated timeline (2018–2025) visualizes the parallel progression of major research themes. Each colored bar represents a thematic focus, with its vertical blue circles scaled to indicate the theme’s annual publication volume. The intentional overlap of elements highlights periods of co-occurring activity and conceptual synergy, depicting the field’s collaborative growth.

To comprehensively address the developmental trajectory of AI in OT diagnosis, we synthesized the methodological evolution across four conceptual frameworks (Figure 3, Figure 4, Figure 5 and Figure 6) that visualize both chronological progression and thematic shifts in research focus. To synthesize the diverse approaches across the 22 included studies, Table 1 presents a condensed overview of key methodological trends, with the complete expanded table available in Supplementary Materials (Supporting Information Table S2).

Figure 6 illustrates the chronological evolution of AI research in Ocular Toxoplasmosis diagnosis from 2018 to 2025, revealing two parallel narratives: quantitative growth evidenced by vertical clusters of blue circles representing annual publication volume, and qualitative advancement through color-coded methodological milestones. The visualization clearly shows the field’s accelerated growth particularly from 2023 onward, with progression beginning with foundational technical validations (2018–2019: protozoa detection, basic CNNs), advancing through methodological diversification (2020–2022: instance segmentation, trust analysis, mobile health), and reaching current sophistication (2023–2025: public datasets, AutoML, hybrid architectures) with enhanced clinical focus.

**Figure 7:**
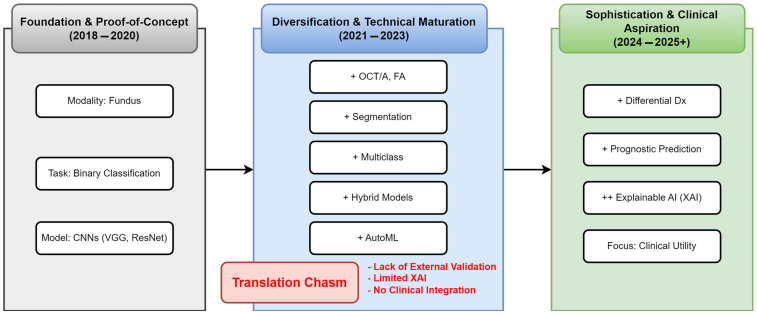
Evolution of Research Focus and The Translational Chasm. The horizontal timeline (→) depicts chronological progression across three research eras. The ‘+’ symbols denote new methodological capabilities introduced in each era, while ‘++’ indicates a significantly intensified focus on specific capabilities. The “Translational Chasm” highlights the persistent gap between technical innovation and clinical implementation.

Figure 7 conceptualizes the methodological progression across three distinct developmental eras, highlighting the emerging ‘Translational Chasm’—a persistent gap between technical innovation and clinical implementation. The *Technical Foundation era* (2018–2020) established core capabilities using fundus photography for binary classification, dominated by technical validation studies. The *Methodological Diversification era* (2021–2023) introduced advanced imaging modalities, complex analytical tasks, and sophisticated architectures, with increased emphasis on clinical relevance and explainability. The current *Clinical Aspiration era* (2024–2025+) focuses on differential diagnosis, prognostic prediction, and enhanced explainability, showing growing focus on external validation and clinical application. However, this evolution reveals that the persistent gap between technical capability and clinical implementation remains the primary barrier to real-world impact, characterized by insufficient external validation and limited clinical integration.

**Figure 8:**
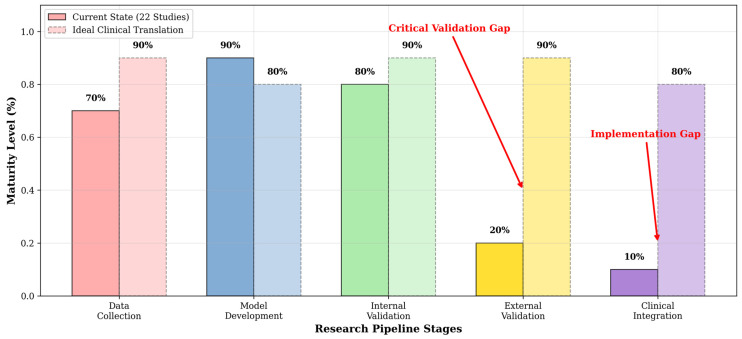
Conceptual Framework: Current vs. Ideal State of AI Research for OT Diagnosis. For each research stage, the left bar represents the “Current State”, derived from an analysis of 22 existing studies. The right bar represents the “Ideal Clinical Translation”, depicting the target maturity required for successful clinical adoption. The red arrow indicates the significant Critical Validation Gap between the Current State and the Ideal Clinical Translation, particularly in the stages of External Validation (20% vs. 90%) and Clinical Integration (10% vs. 80%).

Figure 8 presents a conceptual framework benchmarking current methodological maturity against ideal clinical translation standards, based on our synthesis of 22 studies. The analysis reveals advanced technical capabilities in model development (90% maturity) and internal validation (80%), but critical deficiencies in external validation (20%) and clinical integration (10%). This framework identifies two primary gaps: the ‘Critical Validation Gap’ in external validation and the ‘Implementation Gap’ in clinical integration, which represent the most significant barriers to clinical adoption. The ideal state requires rebalancing efforts toward rigorous external validation (target: 90%) and clinical workflow integration (target: 80%), representing the essential pathway from technical innovation to clinical utility.

**Figure 9:**
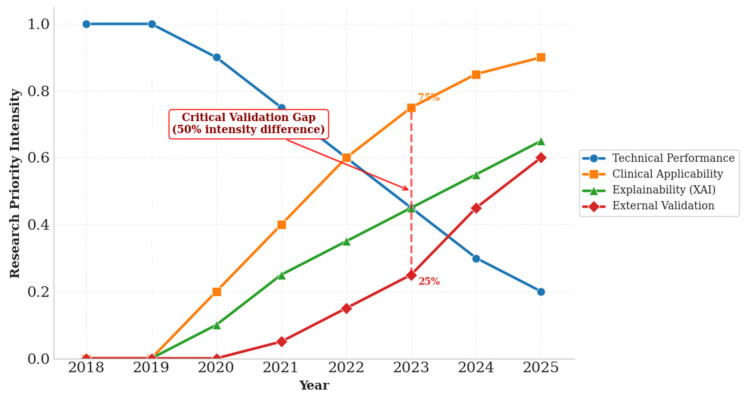
Temporal Evolution of Research Priorities in AI for Ocular Toxoplasmosis. The dashed vertical line at 2023 marks the “Critical Validation Gap”—a 50% intensity difference between Clinical Applicability (75%) and External Validation (25%). This gap highlights the field’s key translational challenge, aligning with the finding that only 9% of studies performed adequate external validation.

Figure 9 visualizes the temporal evolution of research priorities across 22 OT AI studies (2018–2025), elucidating translational challenges through empirical analysis of actual study characteristics from Table 1. The data reveal a clear transition from exclusive technical focus in early years (100% in 2018–2019) toward growing emphasis on clinical applicability and explainability (2021–2023), with recent attention to implementation readiness (2024–2025). The 50% intensity gap between clinical applicability (75%) and external validation (25%) in 2023 underscores the field’s primary translational challenge, consistent with our finding that only 9% of studies conducted proper external validation. While this evolution reflects maturation from pure technical validation toward clinically oriented research, implementation-focused work remains critically underdeveloped. Collectively, these conceptual frameworks demonstrate that despite remarkable technical sophistication, achieving clinically impactful solutions requires deliberate addressing of validation and implementation deficiencies before attaining full implementation capability.

### 3.3. The Data Landscape and Its Challenges

The integration of artificial intelligence (AI) in diagnosing Ocular Toxoplasmosis (OT) is fundamentally constrained by the data used for model development and validation. Our analysis of the 22 included studies reveals a landscape characterized by small, imbalanced, and predominantly single-center datasets, posing significant challenges to the generalizability and clinical translation of these AI models. The descriptive statistics of the datasets are summarized in Table 5.

The descriptive statistics highlight a wide heterogeneity in data availability. The median dataset size was approximately 412 images, but the range was vast (38–5566), with many studies operating on the lower end. For instance, Parra et al. utilized a dataset of 160 images [34], while Cruz-Abrams et al. represented the upper extreme with 5566 images [23]. This scarcity of data is compounded by its source; 14 studies [16,17,19,21,23,24,25,27,30,31,32,33,34,36] relied on datasets from a single institution, underscoring a notable lack of multi-center data. The field’s nascent stage in data sharing is further emphasized by the fact that only two studies [16,31] explicitly used a public dataset (the Ocular Toxoplasmosis Fundus Images Dataset—OTFID).

Fundus photography was the overwhelmingly dominant imaging modality, employed in 18 of the 22 studies [16,17,18,23,24,25,26,27,30,31,32,33,34,36,37] due to its readily accessible and offers a straightforward view of Ocular Toxoplasmosis-related damage. In contrast, more advanced modalities were used sparingly: Optical Coherence Tomography (OCT) was incorporated in only two studies [21] and OCT Angiography (OCTA) in one study [21], primarily for detailed lesion measurement rather than primary AI-driven diagnosis.

A common challenge was class imbalance, explicitly reported or evident in 9 studies [16,17,20,27,30,31,32,33,37]. For example, the widely used OTFID dataset contains 412 images but only 36 examples of active lesions [31]. This imbalance forces researchers to rely heavily on data augmentation techniques, as seen in studies by Ferdous et al. and Zaman et al. [17,30]. While necessary, this method is an imperfect substitute for diverse, real-world data and risks teaching models artifacts instead of true pathological features.

Beyond size and imbalance, several other data-related limitations were frequently cited. The gold standard for labeling was typically manual annotation by expert ophthalmologists, a process used in 14 studies [16,17,18,21,25,26,28,30,31,32,33,34,36,37] that is time-consuming and introduces inter-observer variability. Furthermore, the almost complete absence of external validation on independent datasets was a critical gap, with only two studies [26,28] undertaking this essential step. This raises significant concerns about model overfitting and poor performance in real-world environments. Finally, the lack of explainability (XAI) in 11 of the 22 models [17,18,19,21,23,27,29,30,32,33,35] means that even with high performance metrics, the AI’s decision-making process remains a “black box,” severely limiting clinical trust and adoption [34].

### 3.4. Pathways to Clinical Translation

The assessment of the clinical translation readiness of the included AI studies reveals a significant disparity between high reported performance metrics and the practical steps required for integration into real-world healthcare settings. A critical weakness in validation strategies primarily drives this gap. While the majority of studies (*n* = 16, 72.7%) relied solely on internal validation (e.g., train-test splits), this approach is highly susceptible to overfitting and data leakage, often inflating performance estimates on a model’s native dataset. This creates a ‘generalization paradox,’ where high internal accuracy masks poor performance on data from different populations or clinics. Consequently, the reliance on internal validation alone represents a fundamental methodological limitation. The clinical readiness of a model can only be credibly assessed through external validation on independent datasets, a rigorous step undertaken by only two studies (9.1%) [26,28]. This validation chasm is the most significant barrier to clinical translation identified in this review.

**Figure 10:**
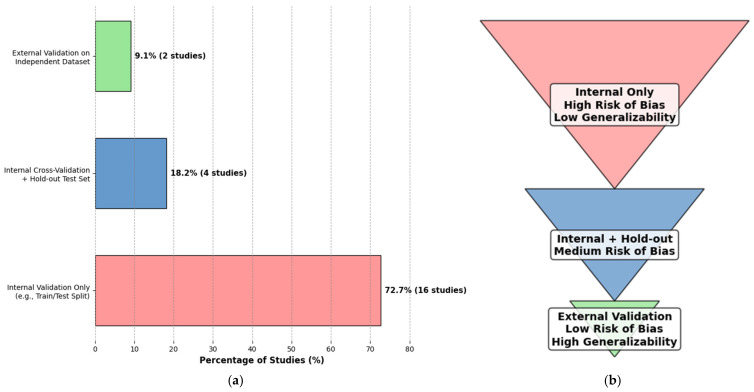
Validation Strategies and Associated Translational Risk in AI Studies for Ocular Toxoplasmosis (*n* = 22). (**a**) The distribution of validation strategies employed across the 22 included studies. (**b**) The correlation between validation strategy and the risk of bias in generalizability estimates, highlighting why external validation is the non-negotiable standard for clinical translation. Color Explanation: Red = High-risk validation (internal only), Blue = Moderate-risk validation (internal + hold-out), Green = Low-risk validation (external, ideal for translation).

Validation strategies employed across the studies were a primary indicator of robustness. As illustrated in Figure 10, the vast majority of studies (*n* = 16, 72.7%) relied solely on internal validation methods, such as a simple hold-out train/validation/test split or k-fold cross-validation. While these methods are a necessary first step, they are highly susceptible to data leakage and overfitting, often inflating performance estimates. A smaller subset of studies (*n* = 4, 18.2%) strengthened their methodology by incorporating both internal cross-validation and a separate internal hold-out test set. Crucially, only two studies (9.1%) conducted proper external validation on a completely independent dataset sourced from a different institution or population [26,28].

This lack of external validation is arguably the most critical weakness, as it leaves the generalizability and reliability of these models in unseen clinical environments largely unproven. The full spectrum of validation rigor and its implications for clinical translation are visualized in Figure 10, which juxtaposes the current distribution of validation strategies with their associated risk of bias. The pyramid structure clearly illustrates why external validation represents the non-negotiable standard for models claiming clinical utility, highlighting the significant validation chasm that currently constrains the field.

Reported model performance was generally high but exhibited a wide range, reflecting differences in datasets, tasks, and model architectures. For binary classification tasks (e.g., healthy vs. OT), accuracy ranged from approximately 87% to 99.2%, sensitivity from 82% to 100%, and specificity from 83% to 98% [24,25,26,30,32]. Area Under the Curve (AUC) metrics, where reported, were also strong, reaching up to 1.00 in one study [24]. However, these impressive figures must be interpreted with caution, given the previously mentioned limitations of small, imbalanced, and internally validated datasets.

A major hurdle for clinical adoption is the lack of explainability. The “black box” nature of complex AI models erodes clinician trust. Our analysis found that only eight studies (36.4%) incorporated any form of Explainable AI (XAI) methodology to interpret model predictions. The most commonly employed technique was Grad-CAM [16], followed by other methods such as Saliency Maps [25], Integrated Gradients [34], and SHAP [22]. The remaining fourteen studies (63.6%) did not include any XAI component, presenting their models without mechanisms to justify their diagnostic decisions to a practicing ophthalmologist.

Finally, the discussion of clinical integration was markedly absent. No study provided a concrete plan or pilot demonstration for integrating the AI tool into existing clinical workflows, such as Picture Archiving and Communication Systems (PACSs) or electronic health records (EHR). Similarly, none addressed the regulatory pathways (e.g., FDA approval, CE marking) required for clinical deployment. The discourse on readiness was consistently tentative, with most studies concluding that their work was a “proof-of-concept” or “promising but requiring further validation” before clinical use [16,17,37].

In conclusion, while the foundational AI research for OT diagnosis demonstrates high potential efficacy, the pathway to clinical translation is underdeveloped. A validation gap currently constrains the field, a transparency deficit due to limited XAI, and a lack of strategic planning for practical implementation and regulatory approval.

### 3.5. AI Research Trends and Trajectories

**Figure 11:**
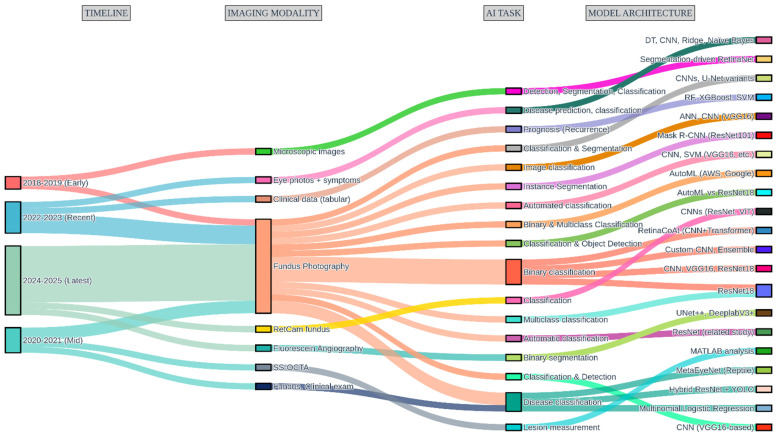
AI Research Pipeline in Ophthalmology.

Figure 11 presents a comprehensive mapping of the evolving research pipeline in artificial intelligence (AI) for Ocular Toxoplasmosis (OT) diagnosis, illustrating the flow from temporal trends through imaging modalities and AI tasks to model architectures. This visualization synthesizes data from 22 included studies and reveals several key developmental patterns in the field.

Temporally, research activity has accelerated markedly from the early period (2018–2019) [20,37] through the most recent phase (2024–2025) [24,26,28], with a progressive diversification of methodological approaches. Fundus photography remains the overwhelmingly dominant imaging modality, forming the foundation for the majority of AI applications [16,17,24,30,31]. However, emerging modalities, including swept-source optical coherence tomography angiography (SS-OCTA) for detailed lesion measurement [21], fluorescein angiography for vascular analysis [29], and structured clinical data for prognostic modeling [22] are gaining traction in more recent, specialized studies.

The analysis of AI tasks demonstrates a clear hierarchy of complexity. Binary classification (e.g., healthy vs. diseased) represents the most common and established application, serving as the entry point for many research efforts [24,25,34]. The field shows clear maturation through the incorporation of more sophisticated tasks such as multiclass classification of disease activity (active vs. inactive) [26,33], precise instance segmentation of lesions [18], and even prognostic prediction of disease recurrence [22]. This evolution reflects a translational trajectory from basic detection toward clinically nuanced applications that address specific diagnostic challenges.

Architecturally, Convolutional Neural Networks (CNNs)—particularly pre-trained backbones like ResNet and VGG16—form the computational backbone of most models, often enhanced through transfer learning to mitigate data scarcity [16,25,36]. The landscape shows increasing architectural innovation with the emergence of hybrid CNN-Transformer models designed to capture both local and global image features [24], automated machine learning (AutoML) platforms that streamline model development [26,28], and specialized architectures for segmentation (U-Net++, DeepLabV3+) [29] and instance detection (Mask R-CNN) [18]. This architectural diversity underscores the field’s technical maturation and active exploration of solutions, while simultaneously highlighting the absence of a singular, universally optimal approach.

Collectively, this pipeline analysis demonstrates both the consolidation of proven methodologies and the strategic exploration of novel approaches. It depicts a field in a crucial transition phase, moving from initial proof-of-concept demonstrations anchored on fundus images toward the development of more sophisticated, clinically integrated diagnostic solutions that leverage multimodal data and address a wider spectrum of clinical tasks.

## 4. Discussion

### 4.1. Summary of Key Findings

This scoping review maps the emergent field of AI for Ocular Toxoplasmosis (OT) diagnosis revealing a significant disconnect between technical promise and clinical readiness. The field is rapidly evolving, with research dominated by proof-of-concept studies that demonstrate the technical feasibility of using deep learning models, primarily CNNs and increasingly hybrid architectures, to automate detection from fundus photos with high reported accuracy [16,24,25], as systematically visualized in Figure 7.

A notable strength is the field’s technical creativity, exploring tasks beyond classification like segmentation and monitoring [21,37] and leveraging AutoML to democratize development [26]. However, these innovations are constrained by a foundational weakness: a data landscape plagued by small, imbalanced, single-center datasets [17,34]. This leads to a critical over-reliance on internal validation that threatens generalizability and clinical applicability. This weakness appears more pronounced in OT research due to its rarity compared to other ophthalmic diseases like diabetic retinopathy [38].

The pathway to the clinic remains underdeveloped, creating a significant translational chasm. Unlike more mature AI fields in ophthalmology, OT research suffers from a stark deficiency in external validation and a neglect of Explainable AI (XAI), which sustains the “black box” problem and erodes clinician trust [22,34]. Furthermore, the literature shows an almost complete absence of discussion on two vital pillars of implementation: integration into clinical workflows and alignment with regulatory pathways.

The implications demand a collaborative paradigm shift. Future research must prioritize the creation of multi-center datasets, mandate external validation, and embed XAI into model development. For clinicians, the current takeaway is that these tools are not yet ready for deployment. Their crucial role is to enable future progress by championing standardized data collection. Closing the gap between potential and readiness will require a concerted, interdisciplinary effort to build transparent, validated, and clinically aligned AI systems.

### 4.2. Interpretation of the Evidence

The evaluation of the AI for OT diagnostic landscape reveals a field defined by a critical tension between compelling technical performance and significant translational barriers, presenting a clear paradox between promise and readiness. While Figure 7 illustrates a promising trajectory of methodological diversification and architectural innovation, this technical progress contrasts sharply with persistent validation gaps.

Consistently high performance metrics strongly support the foundational promise of AI in this domain. Numerous studies report accuracies frequently exceeding 90% [16,17,30], demonstrating that deep learning models can indeed discern the morphological features of OT lesions in fundus photographs. This level of performance aligns with advancements in other ophthalmic AI applications, such as diabetic retinopathy screening [39], and solidifies the technical viability of this approach, justifying continued investment and research.

However, these robust performance figures are entangled in a central paradox: they are predominantly achieved on datasets that are intrinsically insufficient for developing generalizable clinical tools. The field’s overwhelming reliance on small, single-center, and imbalanced datasets [28,37] risks creating a mirage of efficacy. High accuracy in such constrained environments often reflects model overfitting and dataset-specific bias rather than true diagnostic robustness capable of functioning across diverse populations and clinical settings [40,41]. This paradox is particularly acute in OT due to the disease’s relative scarcity, necessitating innovative data strategies like few-shot learning [27].

Beyond data limitations, clinical adoption is severely hampered by a pervasive lack of model transparency. While a minority of studies incorporated explainability methods like Grad-CAM [26,28], the majority presented models as “black boxes” [34]. For clinicians, the inability to understand the reasoning behind an AI’s diagnosis is a fundamental barrier to trust, accountability, and safe integration into the clinical decision-making process [42,43]. The absence of explainability is therefore not merely a technical omission but a critical impediment to regulatory approval and clinical workflow integration.

The most formidable barrier to translation is the profound validation chasm. Although internal validation is common, there is a near-total absence of rigorous external validation on independent, multi-center datasets [22,26]. Furthermore, no study implemented prospective validation in a live clinical setting. This chasm separates promising laboratory results from proven clinical utility, as performance on retrospective data often fails to predict real-world effectiveness [44]. Without this essential step, the true generalizability and readiness of these models remain unknown.

Collectively, these findings illustrate that while technical innovation is robust, the path to clinical impact is constrained by systemic challenges. Future research must undergo a paradigm shift: prioritizing the creation of large, diverse, multi-center datasets, embedding explainability by design, and mandating external and prospective validation as a non-negotiable standard. For clinicians, the current role is to advocate for standardized data collection to build the foundational repositories required for the future development of trustworthy, clinically integrated AI tools.

### 4.3. Implications for Clinical Practice and Research

Artificial intelligence has become an innovative solution that can handle numerous problems that healthcare systems face. AI, using machine learning and deep learning techniques, is capable of scrutinizing intricate data collections such as digital patient files, medical scans, and genetic data to spot trends, forecast how diseases will advance, and advise on the most effective treatment plans. AI also has the potential to promote equity by enabling cost-effective and resource-efficient solutions in resource-limited and remote settings. However, robust ethical and legal structures are crucial for keeping patient details private, making sure data is safe, and setting up who is in charge when choices are made using AI. Obtaining informed consent and complying with regulations, such as the Health Insurance Portability and Accountability Act (HIPAA) and General Data Protection Regulation (GDPR) are vital for keeping data use secure [45].

The current state of AI for OT diagnosis, marked by technical innovation yet translational fragility, necessitates a coordinated roadmap for both researchers and clinicians to bridge the gap between algorithmic potential and tangible patient impact. The research pipeline depicted in Figure 11 shows encouraging diversification, but this must now be channelled toward addressing the critical gaps identified in this review. In previous research, the innovative deep learning system called RetinaCoAt was used to automatically find Ocular Toxoplasmosis in pictures of the retina. The performance of this system, which combines a CNN with an attention mechanism based on transformers, was remarkably high; the model’s ROC score was a flawless 1.00, underscoring its robustness and reliability in distinguishing between healthy and infected cases [24].

#### 4.3.1. For Researchers: A Call for Standardization, Transparency, and Clinical Relevance

To advance beyond proof-of-concept, the research community must address critical gaps through collaborative and rigorous practices. The foremost priority is overcoming data fragmentation by building large, multi-center, publicly available datasets [16,34]. International consortia, modeled on successful initiatives in other ophthalmic fields [39], are essential to aggregate diverse, annotated imaging data. This must be coupled with the development of standardized reporting guidelines to ensure transparency in data demographics, labeling protocols, and validation methods, enabling robust benchmarking and meta-analysis [28].

Furthermore, the research agenda must evolve to reflect clinical complexity. Future work should prioritize AI-assisted differential diagnosis to distinguish OT from other posterior uveitis [26,33], a task of direct relevance to practitioners. Crucially, explainable AI (XAI) must be a foundational component, not an optional feature. The pervasive “black box” problem undermines trust and adoption [34]; integrating techniques like Grad-CAM or SHAP is vital for creating transparent, verifiable tools that meet regulatory and clinical standards [42]. Finally, rigorous external and prospective validation is the non-negotiable bridge to clinical utility. Moving beyond internal benchmarks to studies that assess impact on diagnostic speed and accuracy in real-world settings is imperative for translation [22,44].

#### 4.3.2. For Clinicians: Strategic Engagement with an Evolving Technology

For clinicians, this review underscores the importance of maintaining realistic expectations. The current generation of AI models is investigational tools derived from retrospective data; they are not yet validated for independent clinical use and should be viewed as promising decision-support aids rather than diagnostic replacements [46,47].

However, the future potential is significant, particularly for triaging and supporting diagnosis in resource-limited settings where specialist access is scarce [1]. To realize this potential, clinicians must transition from passive observers to active collaborators in the research ecosystem. This involves advocating for and implementing standardized imaging protocols within clinical practice to generate the high-quality, real-world data necessary for developing robust AI. By engaging with researchers to co-design studies and define clinically meaningful endpoints, clinicians can ensure that these tools are not only accurate but also usable, relevant, and seamlessly integrated into existing workflows [23,24].

In conclusion, the path to clinical integration is a symbiotic one. It demands that researchers prioritize clinical needs through collaboration, explainability, and rigorous validation, while clinicians contribute their expertise to guide development and prepare the groundwork for implementation. Through this shared commitment, AI can evolve from a fragmented collection of prototypes into a reliable, standardized partner in improving patient care for Ocular Toxoplasmosis.

## 5. Limitations

A primary limitation stems from the inherent heterogeneity of the included studies. The substantial variations in technical approaches—including imaging modalities (e.g., fundus photographs, OCT, OCTA), model architectures (from CNNs to Transformers), and task definitions (binary vs. multiclass classification, segmentation)—precluded formal meta-analysis and direct quantitative comparisons between proposed AI systems. This diversity, while illustrative of a nascent and innovative field, limits the ability to draw definitive conclusions regarding optimal technical pathways.

Furthermore, the review’s findings are potentially influenced by selection and publication biases. The overrepresentation of proof-of-concept studies with high performance metrics may reflect a broader trend in AI publications, where studies demonstrating successful outcomes are more likely to be published than those reporting negative results or algorithmic failures. Consequently, the reviewed literature may present an overly optimistic view of real-world model performance and generalizability, a challenge noted across medical AI research [40].

The exclusive reliance on digital imaging data presents another constraint. The reviewed studies focused predominantly on automating image interpretation, largely decoupling the AI task from the broader clinical context. This overlooks the integrative diagnostic process used by clinicians, who synthesize imaging findings with patient history, symptoms, and serological results [48,49]. The current generation of AI models, as captured in this review, does not address this crucial synthesis, limiting the clinical relevance of the findings.

Finally, the dynamic and rapid pace of AI development itself is a limitation. This review provides a systematic snapshot of the literature up to early 2025. Given the field’s velocity, it is certain that new architectures, larger datasets, and novel applications are emerging beyond the scope of this analysis. The inclusion of the most recent studies, some of which are yet to undergo the complete peer-review cycle, was necessary to capture the state-of-the-art, but also introduces an element of uncertainty.

Despite these limitations, this scoping review successfully maps the conceptual territory, key evidence, and critical gaps, establishing an essential foundation for guiding future rigorous and clinically oriented research in this domain.

## 6. Recommendations

To bridge the gap between algorithmic performance and patient care, future research must be guided by the following imperative recommendations:

- Focus on Differential Diagnosis: Move beyond binary (healthy vs. diseased) or simple multiclass (active vs. inactive) classification. Research must prioritize developing AI models that assist in the clinically critical task of differential diagnosis, distinguishing OT from other infectious posterior uveitis, such as acute retinal necrosis, syphilitic retinitis, or fungal chorioretinitis [48]. This aligns with the complex reality faced by ophthalmologists and is where AI support would be most valuable.
- Collaborate to Create Large, Multi-Center Datasets: The most significant bottleneck to progress is data. Isolated efforts will remain insufficient. The field must embrace large-scale collaboration to create curated, multi-center, and publicly available datasets. These datasets must encompass diverse patient demographics, imaging devices, and global regions to capture the full heterogeneity of OT manifestations. International consortia, akin to the PROTON Study Group [22], are essential to pool resources and expertise.
- Prioritize Rigorous External Validation: Internal validation is a necessary but insufficient step. Rigorous external validation on completely independent datasets must become a non-negotiable standard for any study claiming clinical relevance [26,28]. This is the only way to truly assess model generalizability and prevent the field from being misled by over-optimistic performance metrics derived from overfitting.
- Integrate XAI as a Core Component: Explainable AI (XAI) cannot be an optional add-on. To build clinician trust and facilitate adoption, XAI methodologies must be a core, integral component of model development from the outset. Techniques such as Grad-CAM or SHAP should be employed to make the model’s decision-making process transparent, interpretable, and auditable [22,34].
- Conduct Prospective Clinical Trials: The ultimate test of any diagnostic tool is its performance in real-time clinical practice. The field must progress to prospective studies that evaluate AI tools not on retrospective datasets, but embedded within live clinical workflows. These trials should assess critical endpoints beyond accuracy, such as diagnostic speed, clinician confidence, change in management decisions, and ultimately, patient outcomes.
- Integrating AI with the serological test as a common diagnostic tool for toxoplasmosis would be a future innovative tool. Previous research showed the use of Artificial Intelligence (AI) combined with a traditional author-designed questionnaire, coproscopic methods, and serology as a valuable tool in diagnosing human intestinal parasites demonstrated significant advantages, including high accuracy for negative cases and significant time efficiency. Automated data processing, structured database design, and real-time performance metric computation reduced the time required for diagnosis compared to traditional approaches [50].

## 7. Conclusions

Artificial intelligence holds a paradigm-shifting potential to standardize and scale the diagnosis of Ocular Toxoplasmosis. However, translating this potential into clinical impact requires a foundational shift from isolated technical innovation to coordinated, clinically grounded collaboration, prioritizing large and diverse datasets, rigorous external validation, model transparency, and a focus on complex differential diagnosis.

## Figures and Tables

**Table 1:** Summary of included studies on AI models for the diagnosis of Ocular Toxoplasmosis.

Country/Region	Study Focus/AI Task	Imaging/Data Type	Dataset Size (Images/Patients)	AI Model/Architecture Used	Key Performance Metrics
South Korea, Bangladesh, USA [16]	Classification & Segmentation of OT lesions	Retinal Fundus	412 images	CNNs (VGG16, ResNet50, etc.), U-Net variants	Acc > 97%, F1 > 97%, AUC > 0.97, Dice > 0.79
Bangladesh [17]	OT Classification (Active, Inactive, Healthy)	Fundus photography	~700 images (after augment.)	ANN, CNN with VGG16	Accuracy ~85%, F1-score ~0.85
USA, Turkey, Argentina [18]	Instance Segmentation of OT lesions	Fundus Photography	246 images	Mask R-CNN (ResNet101)	Mask IoU > 0.70, AP@50 > 0.80
Sri Lanka [19]	Uveitis Diagnosis & Risk Analysis App	Eye photos + Symptoms	~500+ records	Decision Tree, CNN, Ridge Regression	Accuracy: 87.01% (Disease), 85.41% (Subtype)
Japan & Malaysia [20]	Protozoa Detection in Micrographs	Microscopic images	38 train, 31 test images	Segmentation-driven RetinaNet (ResNet50)	mAP, Precision, Recall
Turkey [21]	Lesion Size Monitoring in OT	SS-OCTA	14 eyes	MATLAB-based analysis (MATLAB R2023a, MathWorks, Natick, MA, USA)	Pixel reduction %, Correlation with VA
Multi-center (Global) [22]	Predicting Uveitis Recurrence	Clinical Data (Tabular)	966 patients	Random Forest, XGBoost, SVM	AUC > 0.80, Accuracy > 75%
USA [23]	Retinoblastoma vs. Pseudo-RB Classification	RetCam Fundus	5566 images	ResNet-101, ViT	Sensitivity 98.6%, Accuracy 97.3%, Specificity 97.0%
Saudi Arabia [24]	OT Diagnosis with Hybrid Model	Retinal Fundus	3659 images (after augment.)	RetinaCoAt (CNN + Transformer)	Accuracy 98%, F1 0.98, AUC 1.00
India [25]	OT Classification (CNN vs. SVM)	Fundus photography	Not specified	CNN, SVM with VGG16, MobileNetV2	Accuracy, Precision, Recall, F1-score
Colombia & Singapore [26]	AutoML for OT Diagnosis & Activity Classification	Color Fundus Photography	681 + 72 (external) images	AutoML (Amazon SageMaker AutoML (2023), AWS, Seattle, WA, USA; Google Cloud AutoML (2023), Google, Mountain View, CA, USA)	Sensitivity 0.97, Specificity 0.98 (AWS); Ext. Val. Acc 87.5%
India [27]	Few-Shot OT Detection	Fundus photography	412 images	MetaEyeNet (Reptile + CNNs)	Accuracy: 75.6–84.76%
Canada [28]	AutoML vs. Custom Model for OT	Fundus photography	304 images	Google AutoML (Google Cloud AutoML (2023), Google, Mountain View, CA, USA) vs. ResNet18	Accuracy 93.5%, Sensitivity 100%
UK, China, Thailand [29]	Segment Leakage & Occlusion in Retinal Vasculitis	Fluorescein Angiography (FA)	463 FA images	UNet++, DeeplabV3+	Dice: Leakage 0.63, Occlusion 0.70
Bangladesh [30]	Automated OT Detection with CNN & Ensemble	Fundus photography	5200 images (after augment.)	Custom CNN, Ensemble (VGG16, VGG19, MobileNet)	Accuracy 97%, AUC high
Paraguay, Spain, Brazil [31]	OT Dataset Description	Fundus photography	412 images	ResNet (related study)	N/A
India [32]	Hybrid Model for OT Detection	Fundus images	291 images	Hybrid ResNet + YOLO	Accuracy 99.2%
Paraguay, Spain [33]	Multiclass OT Classification	Fundus photography	412 images	ResNet18	Accuracy 86.7%, Sensitivity 91.2%
Paraguay, Spain, Brazil [34]	Interpretable DL for OT Diagnosis	Fundus images	160 images	CNN, VGG16, ResNet18	Accuracy, Sensitivity, Specificity, Trust Score
International (Global) [35]	Standardized Classification for Toxoplasmic Retinitis	Fundus, Clinical Exam	803 cases	Multinomial Logistic Regression	Accuracy 92.1–93.3%
Paraguay, Spain, Brazil [36]	ResNet18 for OT Diagnosis	Fundus photography	160 images	ResNet18	Accuracy up to 93.75%, Sensitivity up to 100%
USA, Turkey, Argentina [37]	Automated OT Detection with CNN	Color Fundus Photography	246 patients + controls	Hybrid CNN (VGG16-based)	AUC up to 0.949, Sensitivity 0.919

**Table 2:** Chronological Distribution of Included Studies (2018–2025).

Publication Year	Number of Studies	Cumulative Percentage
2018	1	4.50%
2019	1	9.10%
2020	2	18.20%
2021	4	36.40%
2022	1	40.90%
2023	6	68.20%
2024	6	95.50%
2025	1	100.00%
Total	22	

**Table 3:** Country Affiliation Distribution of Included Studies.

Country	Number of Studies *	Percentage of Studies (*n* = 22)
Paraguay	6	27.30%
United States	5	22.70%
Spain	4	18.20%
Brazil	3	13.60%
India	3	13.60%
Argentina	2	9.10%
Bangladesh	2	9.10%
United Kingdom	2	9.10%
Canada	1	4.50%
China	1	4.50%
Japan	1	4.50%
Malaysia	1	4.50%
Saudi Arabia	1	4.50%
Singapore	1	4.50%
Sri Lanka	1	4.50%
Thailand	1	4.50%
Turkey	1	4.50%
Total Affiliations	34	

Note: * The count represents the number of studies a country is associated with. The sum of affiliations (*n* = 34) exceeds the total number of studies (*n* = 22) due to multi-country collaborations.

**Table 4:** Comparative Analysis of Predominant AI Architectures for Ocular Toxoplasmosis Diagnosis.

Architecture	Prevalence in OT Studies	Key Advantages in OT Context	Key Limitations/Challenges in OT Context	Clinical Translation Implication
CNNs (e.g., VGG16)	Very High	Strong baseline for feature extraction	Prone to overfitting on small datasets	Good for initial proof-of-concept; requires careful regularization and large datasets for robust performance.
		Extensive pre-trained models available	May lose broader contextual information	
		Computationally efficient for transfer learning		
ResNet & Variants	High	Skip connections mitigate vanishing gradients	Still requires significant data for optimal depth	One of the most suitable current architectures for OT, balancing performance and data efficiency.
		Enables effective training of deeper networks	Higher computational cost than simpler CNNs	
		More robust with limited data		
Transformer-Based/Hybrid	Emerging/Low	Potential to model long-range, global dependencies in images	Extremely data-hungry	Not yet practical for most OT applications due to data scarcity; represents a future direction.
		State-of-the-art potential	High computational complexity and cost	
			Poor performance on small datasets	
AutoML Platforms	Emerging/Low	Democratizes model development	“Black box” development process	Useful for rapid prototyping and benchmarking, but may lack the specificity of bespoke models.
		Reduces need for deep ML expertise	Limited customizability for specific clinical tasks	
		Automates hyperparameter tuning	Can be costly	
Instance Segmentation (e.g., Mask R-CNN)	Low	Provides pixel-level lesion localization	Requires extensive, pixel-wise annotated data	High clinical value for monitoring lesion size, but hindered by current data annotation bottlenecks.
		Offers both classification and spatial delineation	Annotation is labor-intensive and expensive	

**Table 5:** Characteristics and Limitations of Datasets in AI Studies for Ocular Toxoplasmosis (*n* = 22).

Characteristic	Findings (*n* = 22 Studies)	Implications & Challenges
Dataset Size (Images)	Median ≈ 412; Range = 38–5566 [23,34]	Wide heterogeneity; most studies operate with small datasets, increasing overfitting risk and limiting model complexity.
Data Source	Single-Center: 14 studies [16,17,19,21,23,24,25,27,30,31,32,33,34,36]	Notable lack of external validation; nascent stage in open science initiatives and data sharing.
	Multi-Center: 4 studies [18,22,26,28]	
	Public Dataset (OTFID): 4 studies [16,27,31,32]	
Imaging Modality	Fundus Photography: 18 studies	Underutilization of advanced modalities (OCT/OCTA) that provide detailed structural and vascular data.
	OCT/OCTA: 2 studies [21,29]	
	Other (Clinical data, Micrographs): 2 studies [20,22]	
Commonly Reported Challenges	Severe Class Imbalance: 12 studies [16,17,20,25,27,30,31,32,33,34,36,37]	Leads to biased models, questions real-world performance, and severely limits clinical trust due to the “black box” problem.
	Lack of External Validation: 20 studies *	
	Limited Use of XAI: 14 studies **	

Note: The ‘*n*’ values for each characteristic are independent and represent the number of studies out of 22 that exhibited each specific characteristic or challenge. * The count for “Lack of External Validation” indicates that only 2 studies [26,28] performed proper external validation. ** The count for “Limited Use of XAI” indicates that only 8 studies incorporated explainable AI methods.

## Data Availability

No new data generated or analyzed.

## Supplementary Materials

The following supporting information can be downloaded at: https://www.mdpi.com/article/10.3390/idr17060148/s1, Table S1: PRISMA 2020 Checklist, Table S2: Summary of included studies on AI models for the diagnosis of Ocular Toxoplasmosis.

## Abbreviations

The following abbreviations are used in this manuscript:

AI	Artificial Intelligence
AUC	Area Under the Curve
CE	European Conformity
CNN	Convolutional Neural Networks
DL	Deep Learning
FDA	U.S. Food and Drug Administration
IoU	Intersection over Union
OCTA	Optical Coherence Tomography Angiography
OT	Ocular Toxoplasmosis
OTFID	Ocular Toxoplasmosis Fundus Images Dataset
PACS	Picture Archiving and Communication System
SHAP	Shapley Additive Explanations
SVM	Support Vector Machine
XAI	Explainable AI
