# Supplementary material for: The Evolution of Artificial Intelligence in Ocular Toxoplasmosis Detection: A Scoping Review on Diagnostic Models, Data Challenges, and Future Directions
Source: Infect Dis Rep. 2025 Dec 8;17(6):148. doi: 10.3390/idr17060148 (PMC12733249; doi:10.3390/idr17060148)
Supplement: Supplementary file 1 [file idr-17-00148-s001.zip › idr-3965424-supplementary.pdf]

The Evolution of Artificial Intelligence in Ocular Toxoplasmosis Detection: A Scoping Review on Diagnostic Models, Data Challenges, and Future Directions.

Dodit Suprianto, Loeki Enggar Fitri, Ovi Sofia, Akhmad Sabarudin, Wayan Firdaus Mahmudy, Muhammad Hatta Prabowo and Werasak Surareungchai

Table S1. PRISMA 2020 Checlist

Section and Topic	Item #	Checklist item	Location where item is reported
TITLE			
Title	1	Identify the report as a systematic review.	Title
ABSTRACT			
Abstract	2	See the PRISMA 2020 for Abstracts checklist.	Abstract section
INTRODUCTION			
Rationale	3	Describe the rationale for the review in the context of existing knowledge.	Introduction, paragraphs 1-3
Objectives	4	Provide an explicit statement of the objective(s) or question(s) the review addresses.	Introduction, last paragraph & Research Questions
METHODS			
Eligibility criteria	5	Specify the inclusion and exclusion criteria for the review and how studies were grouped for the syntheses.	Section 2.3.2: Inclusion and Exclusion Criteria
Information sources	6	Specify all databases, registers, websites, organisations, reference lists and other sources searched or consulted to identify studies. Specify the date when each source was last searched or consulted.	Section 2.2: Identification of Relevant Studies
Search strategy	7	Present the full search strategies for all databases, registers and websites, including any filters and limits used.	Section 2.2
Selection process	8	Specify the methods used to decide whether a study met the inclusion criteria of the review, including how many reviewers screened each record and each report retrieved, whether they worked independently, and if applicable, details of automation tools used in the process.	Section 2.1: Study Design & 2.2: Identification of Relevant Studies
Data collection process	9	Specify the methods used to collect data from reports, including how many reviewers collected data from each report, whether they worked independently, any processes for obtaining or confirming data from study investigators, and if applicable, details of automation tools used in the process.	Section 2.4: Data Charting

Section and Topic	Item #	Checklist item	Location where item is reported
Data items	10a	List and define all outcomes for which data were sought. Specify whether all results that were compatible with each outcome domain in each study were sought (e.g. for all measures, time points, analyses), and if not, the methods used to decide which results to collect.	Section 2.4 & 2.5: Summarizing and Reporting the Results
	10b	List and define all other variables for which data were sought (e.g. participant and intervention characteristics, funding sources). Describe any assumptions made about any missing or unclear information.	Section 2.4 & Table 1
Study risk of bias assessment	11	Specify the methods used to assess risk of bias in the included studies, including details of the tool(s) used, how many reviewers assessed each study and whether they worked independently, and if applicable, details of automation tools used in the process.	Section 2.4 & 2.5; discussed in Results (Table 1, "Gold Standard for Labeling" and "Validation Type")
Effect measures	12	Specify for each outcome the effect measure(s) (e.g. risk ratio, mean difference) used in the synthesis or presentation of results.	Table 1 ("Primary Performance Metrics" column)
Synthesis methods	13a	Describe the processes used to decide which studies were eligible for each synthesis (e.g. tabulating the study intervention characteristics and comparing against the planned groups for each synthesis (item #5)).	Section 2.5 & 2.6
	13b	Describe any methods required to prepare the data for presentation or synthesis, such as handling of missing summary statistics, or data conversions.	Section 2.4 & 2.5
	13c	Describe any methods used to tabulate or visually display results of individual studies and syntheses.	Section 2.5; Table 1, Figures 2-7
	13d	Describe any methods used to synthesize results and provide a rationale for the choice(s). If meta-analysis was performed, describe the model(s), method(s) to identify the presence and extent of statistical heterogeneity, and software package(s) used.	Section 2.5 - Narrative synthesis for scoping review
	13e	Describe any methods used to explore possible causes of heterogeneity among study results (e.g. subgroup analysis, meta-regression).	Section 2.5 - Addressed through thematic analysis in scoping review
	13f	Describe any sensitivity analyses conducted to assess robustness of the synthesized results.	Not applicable to this scoping review methodology
Reporting bias assessment	14	Describe any methods used to assess risk of bias due to missing results in a synthesis (arising from reporting biases).	Section 5. Limitations - Discussed as a limitation
Certainty assessment	15	Describe any methods used to assess certainty (or confidence) in the body of evidence for an outcome.	Not a standard component of a scoping review; implied in Section 4.2 Interpretation of the Evidence
RESULTS			
Study selection	16a	Describe the results of the search and selection process, from the number of records identified in the search to the number of studies included in the review, ideally using a flow diagram.	Section 2.2 & Figure 1: PRISMA Flow Diagram

Section and Topic	Item #	Checklist item	Location where item is reported
	16b	Cite studies that might appear to meet the inclusion criteria, but which were excluded, and explain why they were excluded.	Section 2.2
Study characteristics	17	Cite each included study and present its characteristics.	Table 1: Summary of included studies
Risk of bias in studies	18	Present assessments of risk of bias for each included study.	Table 1 ("Gold Standard for Labeling", "Validation Type"); Section 3.3 (Validation & XAI discussion)
Results of individual studies	19	For all outcomes, present, for each study: (a) summary statistics for each group (where appropriate) and (b) an effect estimate and its precision (e.g. confidence/credible interval), ideally using structured tables or plots.	Table 1 ("Primary Performance Metrics" column)
Results of syntheses	20a	For each synthesis, briefly summarise the characteristics and risk of bias among contributing studies.	Sections 3.1, 3.2, 3.3, 3.4
	20b	Present results of all statistical syntheses conducted. If meta-analysis was done, present for each the summary estimate and its precision (e.g. confidence/credible interval) and measures of statistical heterogeneity. If comparing groups, describe the direction of the effect.	Sections 3.1, 3.2, 3.3 (Narrative synthesis of performance metrics, data characteristics, validation)
	20c	Present results of all investigations of possible causes of heterogeneity among study results.	Sections 3.2 & 4.1 (Discussion of data heterogeneity and its impact)
	20d	Present results of all sensitivity analyses conducted to assess the robustness of the synthesized results.	Not applicable to this scoping review methodology
Reporting biases	21	Present assessments of risk of bias due to missing results (arising from reporting biases) for each synthesis assessed.	Section 5. Limitations - Discussed as a potential limitation
Certainty of evidence	22	Present assessments of certainty (or confidence) in the body of evidence for each outcome assessed.	Section 4.2 Interpretation of the Evidence & 4.3 Implications
DISCUSSION			
Discussion	23a	Provide a general interpretation of the results in the context of other evidence.	Section 4.1 & 4.2
	23b	Discuss any limitations of the evidence included in the review.	Section 4.2 & 5. Limitations
	23c	Discuss any limitations of the review processes used.	Section 5. Limitations
	23d	Discuss implications of the results for practice, policy, and future research.	Section 4.3 Implications & 6. Recommendations
OTHER INFORMATION			

Section and Topic	Item #	Checklist item	Location where item is reported
Registration and protocol	24a	Provide registration information for the review, including register name and registration number, or state that the review was not registered.	Section 2.6: "This scoping review was not registered."
	24b	Indicate where the review protocol can be accessed, or state that a protocol was not prepared.	Section 2.6
	24c	Describe and explain any amendments to information provided at registration or in the protocol.	Section 2.6 - Not applicable as protocol was not registered
Support	25	Describe sources of financial or non-financial support for the review, and the role of the funders or sponsors in the review.	Funding statement
Competing interests	26	Declare any competing interests of review authors.	Conflicts of Interest statement
Availability of data, code and other materials	27	Report which of the following are publicly available and where they can be found: template data collection forms; data extracted from included studies; data used for all analyses; analytic code; any other materials used in the review.	Data Availability Statement

The table format clearly demonstrates that SLR manuscript complies with the PRISMA 2020 quidelines comprehensively, addressing the major elements required for reporting systematic review.

From: Page MJ, McKenzie JE, Bossuyt PM, Boutron I, Hoffmann TC, Mulrow CD, et al. The PRISMA 2020 statement: an updated guideline for reporting systematic reviews. BMJ 2021;372:n71. doi: 10.1136/bmj.n71. This work is licensed under CC BY 4.0. To view a copy of this license, visit https://creativecommons.org/licenses/by/4.0/

Table S2. Summary of included studies on AI models for the diagnosis of Ocular Toxoplasmosis.

Study Characteristics			Population & Data					AI Intervention				Outcomes & Performance		
Country	Study Objective	Study Design	Imaging Modality	Dataset Origin	Dataset Size (Total Images/Patients)	Class Description & Balance	Gold Standard for Labeling	AI Task	Model Type/Architecture	Technical Approach	Explainability (XAI) Method	Primary Performance Metrics	Comparison to Human Experts	Validation Type
South Korea, Bangladesh, USA [16]	To benchmark multiple pre-trained DL frameworks for classification and segmentation of OT lesions in fundus images, and provide a baseline for the OTFID dataset	Experimental benchmark study using transfer learning	Retinal Fundus Photography	OTFID v3 dataset (Hospital de Clínicas & Niños de Acosta Ñú General Pediatric Hospital, Paraguay)	412 fundus images (291 + 121); collected 2018–2021	Healthy vs. Non-healthy; Non-healthy subdivided into Active, Inactive, Active/Inactive; unbalanced classes	Manual segmentation of lesions by ophthalmologists	Classification (Healthy vs Diseased) & Lesion Segmentation	CNNs: VGG16, MobileNetV2, InceptionV3, ResNet50, DenseNet121; U-Net variants with pre-trained encoders	Transfer learning with frozen layers; preprocessing (CLAHE, NLMD, Gaussian smoothing, illumination equalization); augmentation	Grad-CAM for visual interpretability	Acc, Precision, Recall, F1, AUC (classification); Dice, IoU (segmentation)	Not directly compared to clinicians; evaluated against prior AI studies	5-fold cross-validation
Bangladesh [17]	To classify *Ocular Toxoplasmosis* (OT) fundus images into active, inactive, and healthy categories using deep learning models	Experimental study using deep learning models (ANN, CNN) with transfer learning (VGG16)	Fundus photography	Public OT Fundus Images Dataset (OTFID) from "de Clínicas Medical" (291 images) and "Niños de Acosta y Núñez General	412 original images → reduced to 355 (after dropping ambiguous class) → ~700 images after augmentation	Classes: Active lesions (36), Inactive lesions (188), Healthy eyes (131). Severe imbalance addressed via augmentation & normalization	Original clinical diagnosis from ophthalmologists in contributing hospitals	Image classification (diagnosis of OT)	ANN and CNN with pretrained VGG16 backbone	Preprocessing (normalization, grayscale conversion, resizing to 224x224), data augmentation (rotation, flipping, shifting,	None reported (no XAI applied)	Accuracy, Precision, Recall, F1-score, Loss, Confusion Matrix	Not compared directly to human experts	Internal validation using dataset split (before & after augmentation, grayscale conversion)

Study Characteristics			Population & Data					AI Intervention				Outcomes & Performance		
Country	Study Objective	Study Design	Imaging Modality	Dataset Origin	Dataset Size (Total Images/Patients)	Class Description & Balance	Gold Standard for Labeling	AI Task	Model Type/Architecture	Technical Approach	Explainability (XAI) Method	Primary Performance Metrics	Comparison to Human Experts	Validation Type
				Pediatric Hospital" (121 images), published on ScienceDirect						shearing, zooming), transfer learning				
USA, Turkey, Argentina [18]	To develop and fine-tune a Mask R-CNN instance segmentation model for *Ocular Toxoplasmosis* lesions and scars in fundus images, while reducing expert annotation effort	Experimental study with the development of a deep learning model and human-in-the-loop fine-tuning	Fundus Photography (color fundus images)	Three academic institutions in the USA, Turkey, and Argentina	246 images; Train: 180, Test: 31 (10-fold cross-validation)	Classes: Active OT lesions, Inactive lesions, Optic disk, Fovea; annotated and balanced with background patches	Expert ophthalmologists' manual annotations	Instance Segmentation	Mask R-CNN (initialized with MS-COCO pretrained weights, ResNet101 feature extractor for clustering)	Directed fine-tuning with feature clustering, k-means clustering, CLAHE preprocessing, and data augmentation	Not explicitly using XAI; partial interpretability via feature clustering visualization for expert-guided correction	Mask IoU, AP@50, AP@[.5:.05:.95]	Not directly compared; performance benchmarked against baseline model	10-fold cross-validation; experimental evaluation on held-out test set
Sri Lanka [19]	To design a hybrid mobile application that accurately diagnoses and tracks uveitis,	Experimental prototype, machine learning-based mobile health	Eye photographs (for disease subtype detection) + sympto	Collected patient symptom records (~500+) and vision function dataset (~200	~500+ symptom records, ~200 vision function records, sclera images	Symptoms: eye redness, eye pain, light sensitivity, blurred vision, floaters, JIA. Classes: disease vs. non-disease;	Not explicitly stated; clinical scales used (VLSQ-8, VBR-10)	Disease prediction, subtype classification, vision function estimation, risk prediction	Decision Tree (symptom prediction), CNN (disease subtype), Ridge Regression	Image preprocessing (segmentation, filtering), U-Net for sclera segmentat	None explicitly applied; focus on predictive accuracy rather than explainability	Accuracy: Disease prediction 87.01% (Decision Tree), Subtype detection 85.41%	Not compared directly with clinicians; positioned as a supportive tool for	Train-test split (80–90% train, 10–20% test); performance reported with

Study Characteristics			Population & Data					AI Intervention				Outcomes & Performance		
**Country**	**Study Objective**	**Study Design**	**Imaging Modality**	**Dataset Origin**	**Dataset Size (Total Images/Patients)**	**Class Description & Balance**	**Gold Standard for Labeling**	**AI Task**	**Model Type/Architecture**	**Technical Approach**	**Explainability (XAI) Method**	**Primary Performance Metrics**	**Comparison to Human Experts**	**Validation Type**
	performs risk analysis, and supports clinicians	application	m datasets (CSV)	records); SBVPI sclera dataset for segmentation	(size not precisely specified)	subtype classes (anterior, intermediate, posterior, panuveitis). Balance is not fully specified	for validation		(risk analysis), Gaussian Naïve Bayes (vision function)	ion, ML algorithms trained with symptom/ vision data		(CNN), Risk analysis 80.3% (Ridge), Vision function 80% (Naïve Bayes)	clinicians and trainees	confusion matrix
Japan & Malaysia [20]	To automatically detect, segment, and identify protozoans in micrographs	Experimental study with deep learning model development and evaluation	Microscopic images (micrographs of protozoa)	Real patient cases (micrographs of protozoa species)	38 training images with 43 samples, 31 testing images with 74 samples	Eight protozoa species (Giardia lamblia, Iodamoeba butschlii, *Toxoplasma gondii*, Cyclospora cayetanensis, Balantidium coli, Sarcocystis, Cystoisospora belli, Acanthamoeba); highly imbalanced, with some classes having only 1–5 samples	Manual annotation of protozoa instances in micrographs	Object detection, segmentation, and classification of protozoa	Segmentation-driven RetinaNet (based on RetinaNet with ResNet50 backbone + segmentation sub-network)	Transfer learning (COCO pre-trained weights), data augmentation (rotation, color transfer), and life-cycle stage categorization	Class Activation Mapping (CAM) is used to visualize learned features and highlight the model's reliance on background	mAP (mean Average Precision), Precision, Recall	Not directly compared to human experts; baseline comparison against the original RetinaNet.	Internal testing on held-out dataset (31 images, 74 samples)

Study Characteristics			Population & Data					AI Intervention				Outcomes & Performance		
Country	Study Objective	Study Design	Imaging Modality	Dataset Origin	Dataset Size (Total Images/Patients)	Class Description & Balance	Gold Standard for Labeling	AI Task	Model Type/Architecture	Technical Approach	Explainability (XAI) Method	Primary Performance Metrics	Comparison to Human Experts	Validation Type
Turkey [21]	To measure the lesion size reduction in eyes with active Toxoplasma retinochoroiditis during the disease course using SS-OCTA	Retrospective image analysis	Swept-source optical coherence tomography angiography (SS-OCTA)	Dokuz Eylul University Ophthalmology Department (retrospective clinical data)	14 eyes of 13 patients (19 eyes initially, but 5 were excluded)	Single active lesion per eye; balanced across macular (5), peripheral (6), juxtapapillary (3)	Clinical diagnosis by ophthalmologists based on Holland criteria; confirmed with serology	Lesion measurement/ monitoring	MATLAB-based image binarization & pixel analysis	Automated thresholding (Otsu's method), binarization, concentric circle pixel analysis	None explicitly used	Mean black pixel reduction percentage, correlation with visual acuity, statistical significance (p-values)	Not compared directly to human experts, the visual acuity correlation used	Retrospective validation on patient data
Multi-center (Singapore, Indonesia, India, USA, Ethiopia, Germany, Argentina, Colombia) [22]	To develop and evaluate machine learning models for predicting the risk of recurrent uveitis using baseline clinical characteristics, to inform clinical decision-making and risk stratification.	Retrospective analysis using registry data	None (structured tabular clinical data, not imaging)	Ocular Autoimmune Systemic Inflammatory Infectious Study (OASIS) registry	966 patients (1432 eyes)	"Most cases were idiopathic (479 [33.4%])… cohort included infectious, noninfectious, idiopathic, and undetermined etiologies"	Recurrence was assessed by uveitis specialists using the Standardization of Uveitis Nomenclature criteria.	Prognosis: prediction of recurrent vs. non-recurrent course	Random Forest, eXtreme Gradient Boosting (XGBoost), Support Vector Classifier (RBF kernel)	Preprocessing (imputation, normalization, encoding) + bivariate feature selection + five-fold stratified cross-validation + grid search	SHapley Additive exPlanations (SHAP)	Accuracy, sensitivity, specificity, ROC-AUC, precision, recall, F1-score	No direct comparison with clinicians (focus on model performance)	Five-fold cross-validation + hold-out test set (80/20 split, stratified)

Study Characteristics			Population & Data					AI Intervention				Outcomes & Performance		
**Country**	**Study Objective**	**Study Design**	**Imaging Modality**	**Dataset Origin**	**Dataset Size (Total Images/Patients)**	**Class Description & Balance**	**Gold Standard for Labeling**	**AI Task**	**Model Type/Architecture**	**Technical Approach**	**Explainability (XAI) Method**	**Primary Performance Metrics**	**Comparison to Human Experts**	**Validation Type**
USA [23]	To develop and evaluate ML algorithms for distinguishing retinoblastoma from pseudo-retinoblastoma using RetCam fundus images	Single-institution, retrospective observational study (MSKCC IRB-approved)	RetCam fundus imaging	Memorial Sloan Kettering Cancer Center (Ophthalmic Oncology Service)	5,566 images (2,882 retinoblastoma, 1,970 pseudo retinoblastoma, 804 normal pediatric eyes)	Balanced splits: 80% training, 10% validation, 10% testing; pseudo-RB included diverse benign mimics (Coats disease, PHPV, cataract, etc.)	Clinical diagnosis via ophthalmic oncology experts at MSKCC	Classification: differentiate retinoblastoma vs pseudo-retinoblastoma	Deep CNNs (ResNet-18/34/50/101/152) and Vision Transformer (ViT)	Transfer learning, fine-tuning with augmentation (random flip, padding, resize 224×224), SGD optimizer	None applied in this study; XAI methods like LIME/SHAP were only referenced from other works	Best ResNet-101: Sensitivity 98.6%, Accuracy 97.3%, F1 97.7%; ResNet-152: Specificity 97.0%, Precision 97.0%	Not compared directly to human experts; contextualized as aiding ophthalmologists	Internal validation with 80/10/10 split (train/val/test)
Saudi Arabia [24]	To propose RetinaCoAt, a hybrid CNN-Transformer model for automated diagnosis of *Ocular Toxoplasmosis*, integrating local and global retinal features	Experimental deep learning study	Retinal Fundus Photography	Two hospitals: Hospital de Clínicas Medical Center (2018–2020, 291 images), Niños de Acosta Ñu Pediatric Hospital (2021, 121 images)	Dataset 1: 603 original images (132 healthy, 33 active, 187 inactive, 59 mixed, 192 others). After augmentation (Dataset 2): 3,659 images	The original dataset was imbalanced across multiple categories, so it was restructured into binary classes (Healthy vs Unhealthy). Augmentation balanced dataset (1320 healthy, 2339 unhealthy, plus	Clinical diagnosis and ophthalmological fundus examination (supported by serology and PCR where applicable)	Binary classification (Healthy vs Unhealthy eyes)	RetinaCoAt: Hybrid CoAtNet combining CNN + Transformer with residual connections, MBConv blocks, and multi-head relative attention	Convolution for local feature extraction + Transformer-based global attention; preprocessing with normalization and augmentation; AdamW optimizer;	Not explicitly applied; potential for future explainable AI visualizations suggested in discussion.	Accuracy (98%), Precision (0.98), Recall (0.98), F1-score (0.98), ROC AUC = 1.00	Compared against VGG16 (96.87%), CNN (95%), ResNet (93.75%), AutoML (93.5%), Google Cloud AutoML (84.8%) – RetinaCoAt outperformed all	Train/validation/test split with stratified sampling; early stopping, cross-validation-like monitoring

Study Characteristics			Population & Data					AI Intervention				Outcomes & Performance		
Country	Study Objective	Study Design	Imaging Modality	Dataset Origin	Dataset Size (Total Images/Patients)	Class Description & Balance	Gold Standard for Labeling	AI Task	Model Type/Architecture	Technical Approach	Explainability (XAI) Method	Primary Performance Metrics	Comparison to Human Experts	Validation Type
					(binary classes)	validation/test sets)				stratified sampling				
India [25]	To develop and evaluate automated classification of *Ocular Toxoplasmosis* fundus images using CNN and SVM to improve diagnosis	Experimental comparative study (CNN vs. SVM with transfer learning)	Fundus photography	Various clinical settings (fundus datasets, including infected and healthy eyes)	Not explicitly reported; dataset split into training, validation, and test sets.	Two classes: infected eye vs. healthy eye; class imbalance addressed with oversampling/weighting	Clinical diagnosis/fundus image labeling by ophthalmologists (noted as standard reference)	Automated classification of *Ocular Toxoplasmosis*	CNN (baseline) and SVM with VGG16, MobileNetV2, and InceptionV3 feature extractors	Transfer learning, stratified sampling, hyperparameter tuning (Adam/SGD, dropout, batch normalization, learning rate scheduling)	Class Activation Maps, Saliency Maps	Accuracy, precision, recall, F1-score	Compared indirectly, this highlights the reduction of inter-observer variability versus subjective manual diagnosis.	Internal validation on train/val/test split; external dataset testing suggested
Colombia & Singapore [26]	To evaluate the performance of AutoML models in diagnosing *Ocular Toxoplasmosis* (OT) and	Cross-sectional, retrospective, and validation study	Color fundus photography (Optos 200° FOV, ZEISS Visuca	Two Colombian referral centers (2013–2021) and an open-source dataset	Colombian dataset: 1,185 images from 130 patients; Cardozo dataset: 412	Active OT (yellowish-white ill-defined lesions, ± vitritis); Inactive OT (hyperpigmented/gray well-	Labels confirmed by uveitis specialist (30+ years of experience), double-checked,	Binary classification (OT vs no OT); Multiclass classification (Active, Inactive, No OT)	AutoML (AWS S3, Google Cloud Vertex AI); CNN backbone automated	Data split 70/20/10, 5-fold cross-validation, automated hyperparameter	No explicit XAI method reported (focus on AutoML pipeline, no visualization/explainability outputs described)	AWS binary: Sensitivity 0.97, Specificity 0.98, AUPRC 1.00; Google	Compared indirectly: clinical interpretation of fundus by uveitis specialists ranges	Internal validation (70/20/10 split, k-fold); External validation using ASRS Image Bank (72 images)

Study Characteristics			Population & Data					AI Intervention				Outcomes & Performance		
**Country**	**Study Objective**	**Study Design**	**Imaging Modality**	**Dataset Origin**	**Dataset Size (Total Images/Patients)**	**Class Description & Balance**	**Gold Standard for Labeling**	**AI Task**	**Model Type/Architecture**	**Technical Approach**	**Explainability (XAI) Method**	**Primary Performance Metrics**	**Comparison to Human Experts**	**Validation Type**
	classifying its inflammatory activity from fundus photographs		m 224 45° FOV, RGB/dual-channel)	(Cardozo et al. 2018–2021, Argentina); external validation from ASRS Image Bank	images; After curation: 681 usable images; External validation: 72 images	defined, no vitritis); No OT (no lesions, with/without Tg serology). Final: 102 active, 164 inactive, 133 no OT (Cardozo) + Colombian curated set	only images with high confidence included			tuning, early stopping, regularization		Cloud binary: Sensitivity 0.82, Specificity 0.91, AUPRC 0.91; Multiclass F1: 0.88 (AWS, Kappa 0.81) and 0.88 (Google, Kappa 0.82); External validation accuracy 87.5% (binary) and 80.3% (multiclass)	from Sensitivity 71–96%, Specificity 58–100% (Stanford MR et al., 2002); AutoML showed higher reproducibility and consistency	
India [27]	To develop and evaluate a Reptile meta-learning approach (MetaEyeNet) for *Ocular Toxoplasmosis* detection	Experimental study using fundus image classification with a	Fundus photography	*Ocular Toxoplasmosis* Fundus Images Dataset (developed by ophthalmologists, 2	412 images	Healthy vs Diseased (Inactive, Active, Active/Inactive); class imbalance present	Expert ophthalmologists' annotation	Disease classification (*Ocular Toxoplasmosis* detection)	CNN (ResNet50, VGG-16, Inception V3) with Reptile Meta-Learning	Preprocessing (CLAHE, denoising, normalization, GAN augmentation) + Reptile	Not explicitly applied	Accuracy: VGG-16 (75.6%), ResNet50 (80%), Inception V3 (84.76%)	Not compared directly	Few-shot validation: 5-way 20-shots, 2000 meta-iterations

Study Characteristics			Population & Data					AI Intervention				Outcomes & Performance		
Country	Study Objective	Study Design	Imaging Modality	Dataset Origin	Dataset Size (Total Images/Patients)	Class Description & Balance	Gold Standard for Labeling	AI Task	Model Type/Architecture	Technical Approach	Explainability (XAI) Method	Primary Performance Metrics	Comparison to Human Experts	Validation Type
	using few-shot learning with limited fundus image data	meta-learning approach		medical centers)					(MetaEyeNet)	gradient-based meta-learning				
Canada [28]	To assess the performance of AutoML in detecting and localizing *Ocular Toxoplasmosis* (OT) lesions in fundus images compared to expert-designed models	Retrospective experimental study using AutoML and bespoke DL models	Fundus photography	Public dataset (Hospital de Clínicas, Asunción, Paraguay)	304 images (187 OT, 117 normal)	Binary classes: OT (62%), Normal (38%)	Labels by experienced ophthalmologists with access to clinical history and ancillary tests	Binary classification (OT vs normal) and object detection (lesion localization)	AutoML (Google Cloud AutoML Vision) vs bespoke deep learning (ResNet18)	Automated ML pipeline with hyperparameter optimization, single-label classification + object detection	Saliency maps (XRAI – eXplanation with Ranked Area Integrals)	Binary model: AuPRC 0.945, Sensitivity 100%, Specificity 83%, Accuracy 93.5%. Object detection: AuPRC 0.600, Precision 93.3%, Recall 56%	Comparable to bespoke expert-designed models (Parra et al.: Sensitivity 94%, Specificity 86%, Accuracy 91%)	Internal validation (AutoML split 80/10/10) + External validation (30 fundus images: 15 normal, 15 OT, all correctly classified)
UK, China, Thailand [29]	To develop deep learning models to segment retinal vascular leakage and occlusion in RV using FA images	Retrospective, multi-center dataset study	Fluorescein Angiography (FA)	St. Paul's Eye Unit (UK) & Xiamen Eye Center (China)	463 FA images from 82 patients (74 right eyes, 69 left eyes, 94 episodes)	340 leakage images, 144 occlusion images; imbalance addressed by binary segmentation	Manual annotation by ophthalmology experts using ImageJ Labkit	Binary segmentation of leakage & occlusion	UNet++, DeeplabV3+, UNet (with ResNet50 & EfficientNet-B3 backbones)	CLAHE enhancement/no-CLAHE, data augmentation, Dice loss, trained on PyTorch	None explicitly applied for XAI	Dice score: leakage 0.6279, occlusion 0.6992 (95% CI reported)	Compared to prior clinician Dice score (0.483–0.572 in uveitis studies), the model outperformed	Train/validate/test split (60:20:20); external validation across Optos & Heidelberg platforms

Study Characteristics			Population & Data					AI Intervention				Outcomes & Performance		
Country	Study Objective	Study Design	Imaging Modality	Dataset Origin	Dataset Size (Total Images/Patients)	Class Description & Balance	Gold Standard for Labeling	AI Task	Model Type/Architecture	Technical Approach	Explainability (XAI) Method	Primary Performance Metrics	Comparison to Human Experts	Validation Type
													clinicians in leakage segmentation	
Bangladesh [30]	To develop and evaluate CNN and ensemble deep learning models for automated detection of *Ocular Toxoplasmosis*	Experimental study with model development and evaluation	Fundus photography	Hospital de Clínicas (Asunción, Paraguay) & Niños de Acosta Ñu General Pediatric Hospital	291 images (Hospital de Clínicas) + 121 images (Niños de Acosta Ñu) = 412 raw images; expanded to 5200 images after augmentation	Healthy vs. Unhealthy (binary); Unhealthy further divided into active, inactive, active-inactive (imbalanced originally, balanced via augmentation)	Lesion masks segmented and verified by a professional ophthalmologist	Classification (multiclass → binary: healthy vs. unhealthy)	Custom CNN, Ensemble of VGG16, VGG19, MobileNet	Data augmentation (resize, rotation, flipping, Gaussian noise), binary classification, ensemble learning	Not explicitly applied; focus was on accuracy, interpretability, and transparency rather than XAI methods.	Accuracy (97% custom CNN, 96% ensemble), Precision, Recall, F1-score, AUC	No direct human expert benchmarking reported; comparison against pretrained DL models (VGG16/19, MobileNet)	Train/validation split (50 epochs); internal dataset evaluation
Paraguay, Spain, Brazil [31]	To researchers working on ophthalmic image analysis, the dataset will be highly beneficial for applying artificial intelligence	Data article / Dataset description study	Fundus photography (VISUCAM 500, ZEISS; Pictor Plus Portable Ophthalmic	Hospital de Clínicas (Paraguay); Niños de Acosta Ñú General Pediatric Hospital (Paraguay)	412 images: 291 adults (2018–2020) + 121 children (2021)	Healthy (131), Inactive (188), Active (36), Active/Inactive (57)	"The dataset was developed by three ophthalmologists with expertise in toxoplasmosis	Automatic classification of *Ocular Toxoplasmosis* lesions	A related study used Residual Neural Networks (ResNet)	Supervised classification and manual segmentation with Label Studio	Not explicitly reported in the dataset paper	Not applicable in the dataset description	Not applicable in the dataset description	Open-access dataset with heterogeneous sources and manual segmentation

Study Characteristics			Population & Data					AI Intervention				Outcomes & Performance		
Country	Study Objective	Study Design	Imaging Modality	Dataset Origin	Dataset Size (Total Images/Patients)	Class Description & Balance	Gold Standard for Labeling	AI Task	Model Type/Architecture	Technical Approach	Explainability (XAI) Method	Primary Performance Metrics	Comparison to Human Experts	Validation Type
	techniques in the automatic detection of toxoplasmosis chorioretinitis.		Camera)				detection using fundus images."							
India [32]	To improve accuracy in detecting Toxoplasmosis chorioretinitis using a hybrid deep learning approach model	Experimental study with a hybrid deep learning approach	Fundus images	Public dataset: *Ocular Toxoplasmosis Fundus Images Dataset*	291 fundus images (2124×2056 px, resized to 256×256)	Diseased: 279, Healthy: 132 (imbalanced classes)	Not explicitly stated; the dataset is annotated for disease vs. healthy classification.	Disease classification (Toxoplasmosis chorioretinitis detection)	Hybrid ResNet + YOLO classifier	Fitness Sorted-Shark Smell Optimization (FS-SSO) for parameter tuning	Not discussed (qualitative interpretability mentioned, but no formal XAI used)	Accuracy (99.2%), Precision, Recall, F1-score	Compared against DL models (VGG16, AlexNet, Inception, Xception, SqueezeNet); hybrid model outperformed	Train-validation-test split with experimental comparison
Paraguay, Spain [33]	To investigate the application of a deep learning model for multiclass classification of *Ocular Toxoplasmosis* (OT) from fundus images (healthy, active,	Experimental study using transfer learning with a fundus image dataset	Fundus photography	Hospital de Clínicas (Asunción, Paraguay); Hospital General Pediátrico Acosta Ñu (Paraguay)	412 fundus images (132 healthy, 91 active, 190 inactive)	Three classes: Healthy (132), Active OT (91), Inactive OT (190)	Manual segmentation and labeling by ophthalmologists	Multiclass classification (healthy vs active vs inactive OT lesions)	Residual Neural Network (ResNet18, pretrained on ImageNet)	Transfer learning, fine-tuning, data augmentation (flips, rotations, normalization), early stopping, optimizer tuning	Not reported	Accuracy, Sensitivity, Specificity (best: 0.867, 0.912, 0.980 with SGD)	Not directly compared with human experts, but aligned with UK diabetic retinal screening benchmarks (≥80% sensitivity,	Train/validation/test split (70/10/20%)

Study Characteristics			Population & Data					AI Intervention				Outcomes & Performance		
**Country**	**Study Objective**	**Study Design**	**Imaging Modality**	**Dataset Origin**	**Dataset Size (Total Images/Patients)**	**Class Description & Balance**	**Gold Standard for Labeling**	**AI Task**	**Model Type/Architecture**	**Technical Approach**	**Explainability (XAI) Method**	**Primary Performance Metrics**	**Comparison to Human Experts**	**Validation Type**
	inactive lesions)												≥95% specificity)	
Paraguay, Spain, Brazil [34]	To propose a methodology that evaluates DL models for *Ocular Toxoplasmosis* (OT) diagnosis based on interpretability and trust	Experimental study using a retrospective dataset and DL model evaluation	Fundus images	Hospital de Clínicas, Asunción, Paraguay	160 fundus images	Healthy vs. OT (active lesions, inactive scars); balanced samples across classes	Manual segmentation by ophthalmologists (fundus lesion masks)	Binary classification (Healthy vs. OT diagnosis)	CNN (vanilla), VGG16, ResNet18	Transfer learning (VGG16, ResNet18), SGD optimization, data augmentation (flips, rotations), cross-entropy loss	Integrated Gradients (IG) attribution	Accuracy, sensitivity, specificity, trust score	Not directly compared; focus on interpretability for ophthalmologists' trust.	Split: Training 70%, Validation 10%, Test 20%
International (USA, Brazil, France, UK, Australia) [35]	To determine standardized classification criteria for toxoplasmic retinitis using machine learning across multiple infectious posterior uveitides/panuveitides	Multi-center, retrospective cohort with machine learning analysis	Fundus photography, ophthalmic examination	International SUN preliminary & final database (76 investigators worldwide)	803 cases total; 174 cases of toxoplasmic retinitis	Unifocal/paucifocal vs. multifocal necrotizing retinitis; 88% unilateral; balanced across age/sex; PCR/serology subsets	Supermajority expert consensus diagnosis (>75% agreement) + PCR and/or serology confirmation	Disease classification	Multinomial logistic regression	Machine learning on the training set to minimize misclassification	Not specified (no XAI method used)	Accuracy (92.1% training, 93.3% validation); Misclassification rate (8.2% training, 10% validation)	Compared against expert consensus and other infectious posterior uveitides (ARN, CMV, syphilitic, tubercular uveitis)	Independent validation set (~15% of cases)
Paraguay, Spain,	To investigate whether a pretrained ResNet18 can	Experimental study with	Fundus photography	Hospital de Clínicas,	160 images	Healthy vs. OT (active lesions / inactive scars);	Ophthalmologists manually segmented	Binary classification (OT vs. healthy)	Residual Neural Network (ResNet18,	Transfer learning with fine-tuning;	None explicitly applied; focus was on classification	Accuracy (up to 93.75%), Sensitivit	Not directly compared with	Train/Validation/Test split (70/10/20);

Study Characteristics			Population & Data					AI Intervention				Outcomes & Performance		
**Country**	**Study Objective**	**Study Design**	**Imaging Modality**	**Dataset Origin**	**Dataset Size (Total Images/Patients)**	**Class Description & Balance**	**Gold Standard for Labeling**	**AI Task**	**Model Type/Architecture**	**Technical Approach**	**Explainability (XAI) Method**	**Primary Performance Metrics**	**Comparison to Human Experts**	**Validation Type**
Brazil [36]	aid in automatic OT diagnosis using fundus images	deep learning model training and validation	(retinal images)	Asunción, Paraguay		balanced (70/30, 50/50, 30/70 scenarios tested)	lesions and scars to define labels		pretrained on ImageNet)	tested different optimizers (SGD, Adam, RMSProp)	performance (no XAI methods mentioned)	y (up to 100%), Specificity (up to 92.9%)	experts, but intended as a decision-support tool for ophthalmologists	tested on balanced and imbalanced subsets
USA, Turkey, Argentina [37]	To develop an automated CNN-based approach to detect *Ocular Toxoplasmosis* (OT) from fundus images	Experimental study using retrospective fundus datasets with CNN model training and evaluation	Color fundus photography (wide-angled OPTOS, composite, manually composed, regular fundus images)	Byers Eye Institute (Stanford, USA), Gazi University (Turkey), an academic center in Argentina, and the STARE project for healthy controls	246 eyes of 215 OT patients + 66 healthy images	4,461 unhealthy patches (3,445 inactive scars, 1,016 active lesions) vs. 77,284 healthy patches	Annotated and labeled by masked medical graders (green = inactive scars, yellow = active lesions)	Image classification & lesion detection	CNN (VGG16 backbone, hybrid dual-input CNN)	Patch-based sliding window, optic disc isolation, transfer learning with VGG16, hybrid dual-input CNN combining heatmaps & images	Probability heat maps to localize lesions (implicit interpretability)	AUC up to 0.949, Sensitivity 0.919, Specificity 0.93	Not directly compared to experts; first CNN-based OT detection	Dataset split: 70% train, 10% validation, 20% test; cross-sampling ratios (30/70, 50/50, 70/30)
